# Structural Predictions of the SNX-RGS Proteins Suggest They Belong to a New Class of Lipid Transfer Proteins

**DOI:** 10.3389/fcell.2022.826688

**Published:** 2022-02-03

**Authors:** Blessy Paul, Saroja Weeratunga, Vikas A. Tillu, Hanaa Hariri, W. Mike Henne, Brett M. Collins

**Affiliations:** ^1^ Institute for Molecular Bioscience, The University of Queensland, St Lucia, QLD, Australia; ^2^ Department of Cell Biology, University of Texas Southwestern Medical Center, Dallas, TX, United States

**Keywords:** sorting nexin (SNX), regulator of G-protein signaling (RGS), lipid droplets (LDs), AlphaFold2, X-ray crystallography, lipid transfer protein (LTP), phox homology (PX) domain

## Abstract

Recent advances in protein structure prediction using machine learning such as AlphaFold2 and RosettaFold presage a revolution in structural biology. Genome-wide predictions of protein structures are providing unprecedented insights into their architecture and intradomain interactions, and applications have already progressed towards assessing protein complex formation. Here we present detailed analyses of the sorting nexin proteins that contain regulator of G-protein signalling domains (SNX-RGS proteins), providing a key example of the ability of AlphaFold2 to reveal novel structures with previously unsuspected biological functions. These large proteins are conserved in most eukaryotes and are known to associate with lipid droplets (LDs) and sites of LD-membrane contacts, with key roles in regulating lipid metabolism. They possess five domains, including an N-terminal transmembrane domain that anchors them to the endoplasmic reticulum, an RGS domain, a lipid interacting phox homology (PX) domain and two additional domains named the PXA and PXC domains of unknown structure and function. Here we report the crystal structure of the RGS domain of sorting nexin 25 (SNX25) and show that the AlphaFold2 prediction closely matches the experimental structure. Analysing the full-length SNX-RGS proteins across multiple homologues and species we find that the distant PXA and PXC domains in fact fold into a single unique structure that notably features a large and conserved hydrophobic pocket. The nature of this pocket strongly suggests a role in lipid or fatty acid binding, and we propose that these molecules represent a new class of conserved lipid transfer proteins.

## Introduction

The sorting nexins (SNXs) are a large family of proteins with diverse structures and functions. They are found in all eukaryotes and their defining feature is the presence of a phox homology (PX) domain, which most commonly associates with phosphatidylinositol phospholipids (PtdInsPs) to mediate interactions with membranes of the endolysosomal system (Teasdale and Collins, 2012; Chandra and Collins, 2019; Chandra et al., 2019). SNX proteins are typically grouped into sub-families based on the presence of additional functional domains such as membrane tubulating bin/amphiphysin/rvs (BAR) domains, protein interacting SH3 domains, and GTPase activating protein (GAP) domains among many others (Teasdale and Collins, 2012).

**FIGURE 1:**
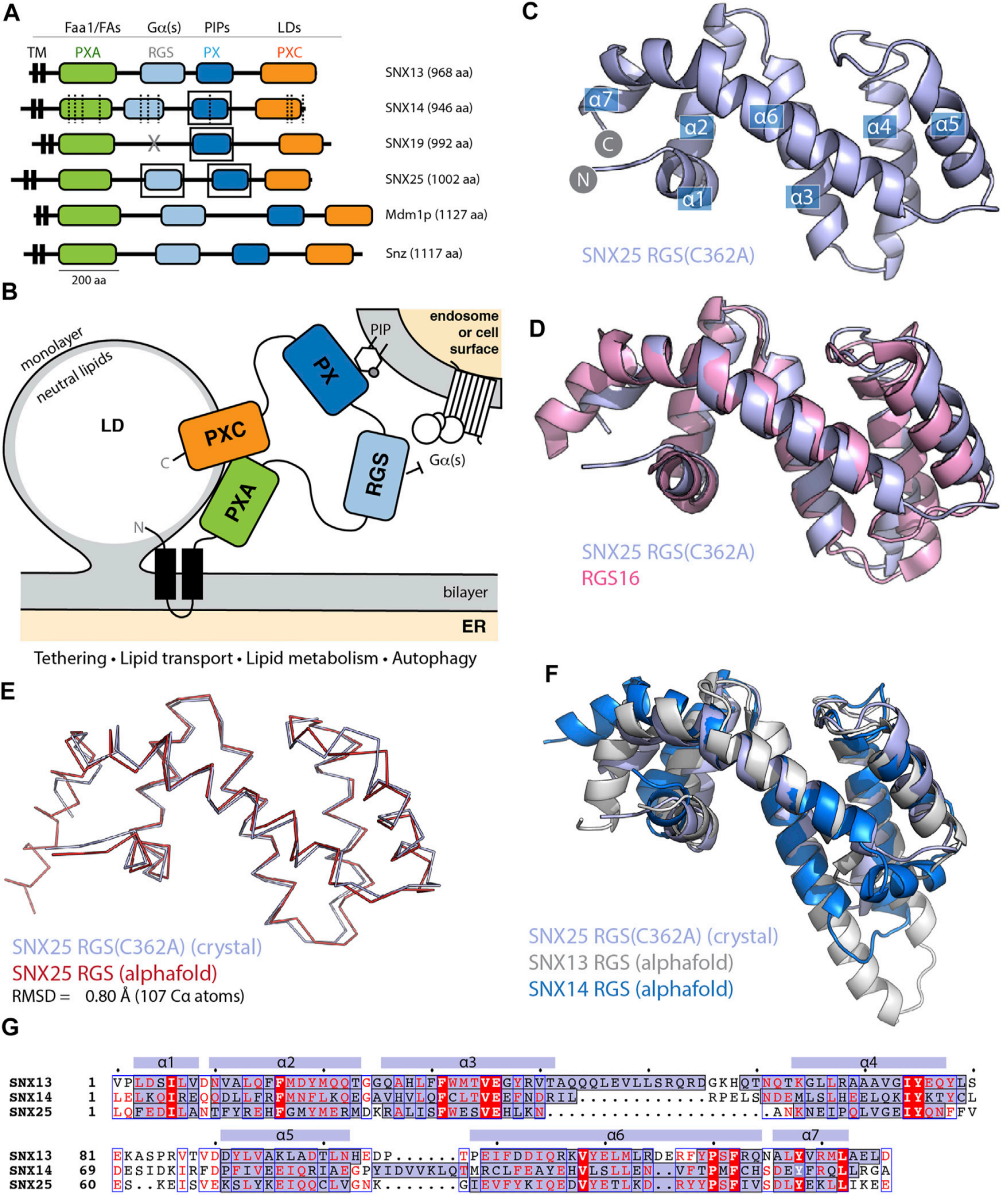
The SNX-RGS protein family. **(A)** Domains of human, *Drosophila*, and *S. cerevisiae* SNX-RGS family members. Putative ligands for each domain are indicated above. SNX14 mutations causing cerebellar ataxia are shown with dashed lines (Thomas et al., 2014; Akizu et al., 2015). Boxes indicate domains with experimental structures determined. **(B)** The current working model for the architecture, localisation and function of the SNX-RGS protein family and their potential roles at the interface between the ER, LDs, and membranes of the endolysosomal system. **(C)** Crystal structure of human SNX25(C362A) RGS domain in cartoon diagram. Helical secondary structure elements are indicated. **(D)** The crystal structure of human SNX25(C362A) RGS domain (light blue) is aligned with the canonical RGS domain of RGS16 (pink) (PDB ID 2IK8) (Soundararajan et al., 2008) and shows the expected topology. **(E)** Cα ribbon diagram showing the overlay of the SNX25(C362A) RGS domain (light blue) with the same sequence predicted in the AlphaFold2 database (red) (Q9H3E2). **(F)** Cartoon representation of SNX25(C362A) RGS domain compared with the SNX13 and SNX14 RGS domains from the AlphaFold2 database (Q9Y5W8; Q9Y5W7). **(G)** Sequence alignment of human SNX13, SNX14 and SNX25 RGS domains based on structural comparisons. The secondary structure of SNX25 derived from its crystal structure is shown schematically above the alignment, while the α-helical regions of the three proteins from their AlphaFold2 predictions are shaded blue within the alignment.

One of the most highly conserved sub-families are the SNX proteins with regulator of G-protein signalling (RGS) domains or SNX-RGS proteins (Amatya et al., 2021; Mas et al., 2014). In humans there are four genes encoding SNX-RGS proteins, SNX13, SNX14, SNX19 and SNX25. Other well-characterised family members include Mdm1 from *Saccharomyces cerevisiae* and Snazarus (Snz) from *Drosophila melanogaster*. These proteins have relatively low sequence similarity across homologues and across species but share a conserved architecture with an N-terminal hydrophobic membrane anchor, and a central RGS domain and PX domain (except for SNX19 orthologues which lack the RGS domain). In addition, the RGS and PX domains are flanked by two sequences referred to as the PX-associated domains PXA and PXC at the N- and C-terminus respectively (Figure 1A). The N-terminal membrane anchor mediates localisation to the endoplasmic reticulum (ER) (Henne et al., 2015; Bryant et al., 2018; Datta et al., 2019; Ugrankar et al., 2019; Saric et al., 2021), and the proteins typically localise to sites of new lipid droplet synthesis and can enhance membrane tethering of the ER to other membrane compartments *via* their PX domains including the vacuole in yeast (Henne et al., 2015; Hariri et al., 2018; Hariri et al., 2019), the plasma membrane in *Drosophila* (Ugrankar et al., 2019) and endolysosomal compartments in mammalian cells (Bryant et al., 2018; Datta et al., 2019; Saric et al., 2021; Lu et al., 2022).

The precise function of the SNX-RGS proteins is still unclear although their mutation or depletion has significant impacts on cellular physiology, particularly with respect to lipid homeostasis and endolysosomal function. Mutations in human SNX14 cause the autosomal-recessive cerebellar ataxia and intellectual disability syndrome SCAR20, with a common cellular phenotype of autophagic structures containing undigested material and increased cholesterol accumulation in endolysosomal organelles (Bryant et al., 2018; Akizu et al., 2015; Thomas et al., 2014). In mice SNX13, SNX14 and SNX25 are all essential for normal development (Bryant et al., 2020; Bult et al., 2019; Zheng et al., 2006), while altering the expression of Snz significantly extends the lifespan of flies (Ugrankar et al., 2019; Suh et al., 2008). Previous work has defined the structures and lipid binding properties of the PX domains of these proteins; the human SNX13 and SNX19 PX domains bind the endosomal lipid PtdIns3P and SNX25 associates with multiple phosphorylated PtdInsP species, while SNX14 has little affinity for PtdInsP lipids due to an altered binding pocket (Chandra et al., 2019; Mas et al., 2014; Ugrankar et al., 2019). The RGS domains of SNX13 and SNX14 associate with the Gα_s_ subunit of trimeric G-proteins (Ha et al., 2015; Zheng et al., 2001). While SNX13 was originally discovered as a GTPase activating protein (GAP) for Gα_s_ (Zheng et al., 2001), SNX14 can bind Gα_s_ but has so far been found to not stimulate its GAP activity (Ha et al., 2015). SNX13 and SNX14 have also been found to associate with the endosomal Rab GTPase Rab9A, although the functional significance of this is unclear (Gillingham et al., 2019). Proteomics studies have identified association of yeast Mdm1 with Faa1 long-chain-fatty-acid--CoA ligase, while *Drosophila* Snz and human SNX14 were associated with fatty acid desaturases Desat1 and SCD1 respectively (Ugrankar et al., 2019; Datta et al., 2020), suggesting a role in lipid and fatty acid regulation. In line with this, members of the SNX-RGS protein family have almost universally been shown to localize to ER-lipid-droplet (LD) contact sites with other organelles (Figure 1B). Yeast Mdm1 localizes to nucleus-LD-vacuole junctions (Henne et al., 2015; Hariri et al., 2019; Castro et al., 2021). Human SNX14 localizes to ER-LD contacts (Datta et al., 2019), and SNX19 has recently been shown to localize to ER-lysosome contacts, where it also contacts LDs (Saric et al., 2021).

A better understanding of the function(s) of the SNX-RGS proteins requires further knowledge of their structures and molecular interactions. Here we have solved the crystal structure of the human SNX25 RGS domain, revealing a typical α-helical RGS fold. Building on this we took advantage of the recently developed AlphaFold2 machine learning (ML)-based structural prediction algorithm (Jumper et al., 2021) and the related AlphaFold2 database (Tunyasuvunakool et al., 2021) to examine the structures of the full-length SNX-RGS proteins. These analyses revealed an intriguing structural fold not previously seen, that is formed by intramolecular association of the distal PXA and PXC domains. A remarkable feature of this structure is the presence of a large hydrophobic cavity. Based on their structures and known localisation and interactions we propose that the SNX-RGS proteins may represent a previously undefined class of lipid transfer proteins (LTPs) that are likely to mediate binding of multiple lipids or other fatty acid derived molecules. Further, using AlphaFold2 we identify an additional yeast PX domain-containing protein Lec1/Ypr097w that contains another likely lipid-binding structure that shares superficial resemblance but is not directly related to the SNX-RGS proteins. This work provides a new understanding of the likely function of the SNX-RGS proteins, presents a structural bioinformatics comparison of novel lipid binding domains across various eukaryotic species, and further demonstrates the utility of new ML-based structure prediction programs such as AlphaFold2 to develop novel structural and functional insights.

## Materials and Methods

### Cloning, Expression and Purification of the SNX25 RGS Domain

The genes encoding the human SNX25 RGS domain (residues 446–569; A0A494C0S0) and the SNX25 RGS(C526A) mutant were synthesised by Gene Universal (USA) and cloned into the expression plasmid of pGEX-4T-2 for bacterial expression with an N-terminal GST tag and thrombin cleavage site was construct was transformed into *Escherichia coli* BL21-CodonPlus (DE3)-RIL cells and plated on lysogeny-broth (LB) agar plates supplemented with ampicillin (0.1 mg/ml). Single colonies were then used to inoculate 50 ml of LB medium containing ampicillin and the culture was grown overnight at 37°C with shaking at 180 rpm. The following day 1 L of LB medium containing ampicillin (0.1 mg/ml) was inoculated using 10 ml of the overnight culture. Cells were then grown at 37°C with shaking at 200 rpm to an optical density of 0.8–0.9 at 600 nm and the protein expression was induced by adding 0.5 mM IPTG (isopropyl-β-D-thiogalactopyranoside). Expression cultures were incubated at 20°C overnight with shaking and the cells were harvested the next day by centrifugation at 4000 rpm for 15 min using a Beckman rotor JLA 8.100. Cell pellets were resuspended in 20 ml (for cell pellet from 1 L culture) of lysis buffer (50 mM HEPES, (pH 7.5), 500 mM NaCl, 5% glycerol, Benzamidine (0.1 mg/ml), and DNAse (0.1 mg/ml)). Resuspended cells were lysed by using the cell disrupter (Constant systems, LTD, UK, TS-Series) and the soluble fraction containing the protein was separated from cell debris by centrifugation at 18,000 rpm for 30 min at 4°C. The soluble fraction was first purified by affinity chromatography using Glutathione Sepharose 4B resin (GE Healthcare) and the GST tag was cleaved on column by incubating the protein with Thrombin (Sigma Aldrich) overnight at 4°C. The next day the protein was eluted using 50 mM HEPES (pH 7.5), 200 mM NaCl. The eluted protein was then concentrated and further purified by gel filtration chromatography (Superdex 75 (16/600), GE Healthcare) using 50 mM HEPES, pH 7.5, 200 mM NaCl, 0.5 mM TCEP (tri(2-carboxyethyl)phosphine) and the fractions corresponding to SNX25 RGS domains were analysed by SDS PAGE.

### Crystallisation and Structure Determination of the SNX25 RGS Domain

Purified proteins were concentrated to 12 mg/ml for crystallisation. Initially 96 well crystallisation screens were set up using the hanging drop method with a Mosquito Liquid Handling robot (TTP LabTech). The optimised diffraction-quality crystals were obtained in 24-well plates using hanging drop method. The SNX25 RGS domain was crystallised in 30% PEG 4000, 0.2 M Sodium acetate, 0.1 M Tris pH 8.5, while the SNX25 RGS(C526A) domain was crystallised in 18% PEG 8000, 0.1 M calcium acetate, 0.1 M sodium cacodylate, pH 6.5. Diffraction data was collected at the Australian Synchrotron MX2 beamline (Clayton, VIC, Australia), integrated with XDS (Kabsch, 2010) and scaled with AIMLESS software (Evans and Murshudov, 2013). The SNX25 RGS structure was solved by molecular replacement using the RGS1 structure as input (PDB ID 2BV1; ^28^) in PHASER (McCoy et al., 2007) and the resulting model was rebuilt and refined by using COOT (Emsley and Cowtan, 2004) and PHENIX (Adams et al., 2010) respectively. The SNX25 RGS(C526A) was solved similarly by molecular replacement using the wild-type SNX25 RGS domain as input.

### Protein Structural Prediction, Modelling and Visualisation

The structural predictions of the full-length proteins from different species including *S. cerevisiae, D. melanogaster, C. elegans, P. falciparum, D. discoideum, A. thaliana, S. pombe, M. janaschii, D. rerio* and *H. sapiens* analysed in this study were obtained from the AlphaFold2 database (https://alphafold.ebi.ac.uk; (Jumper et al., 2021; Tunyasuvunakool et al., 2021)). To generate predicted models of human SNX25 including the previously annotated transmembrane domain (Mas et al., 2014; Lauzier et al., 2021) and the *S. cerevisiae* Lec1/Ypr097w to assess the flexibility of its PX domain, we used the AlphaFold2 neural-network (Jumper et al., 2021) implemented within the freely accessible ColabFold pipeline (Mirdita et al., 2021). For each modelling experiment ColabFold was executed using default settings where multiple sequence alignments were generated with MMseqs2 (Mirdita et al., 2019) and structural relaxation of final peptide geometry was performed with Amber (Hornak et al., 2006) to generate three models per structure. Sequence conservation was mapped onto the modelled structures with Consurf (Ashkenazy et al., 2016). Interior cavities of proteins were mapped and their volumes were calculated using POcket-CAvity Search Application (POCASA)(Yu et al., 2010), with default settings of 2 Å for probe radius and 1 Å for the grid size. All structural images were made with Pymol (Schrodinger, USA; https://pymol.org/2/).

### Localisation of SNX25 in A431 Cells

**FIGURE 2:**
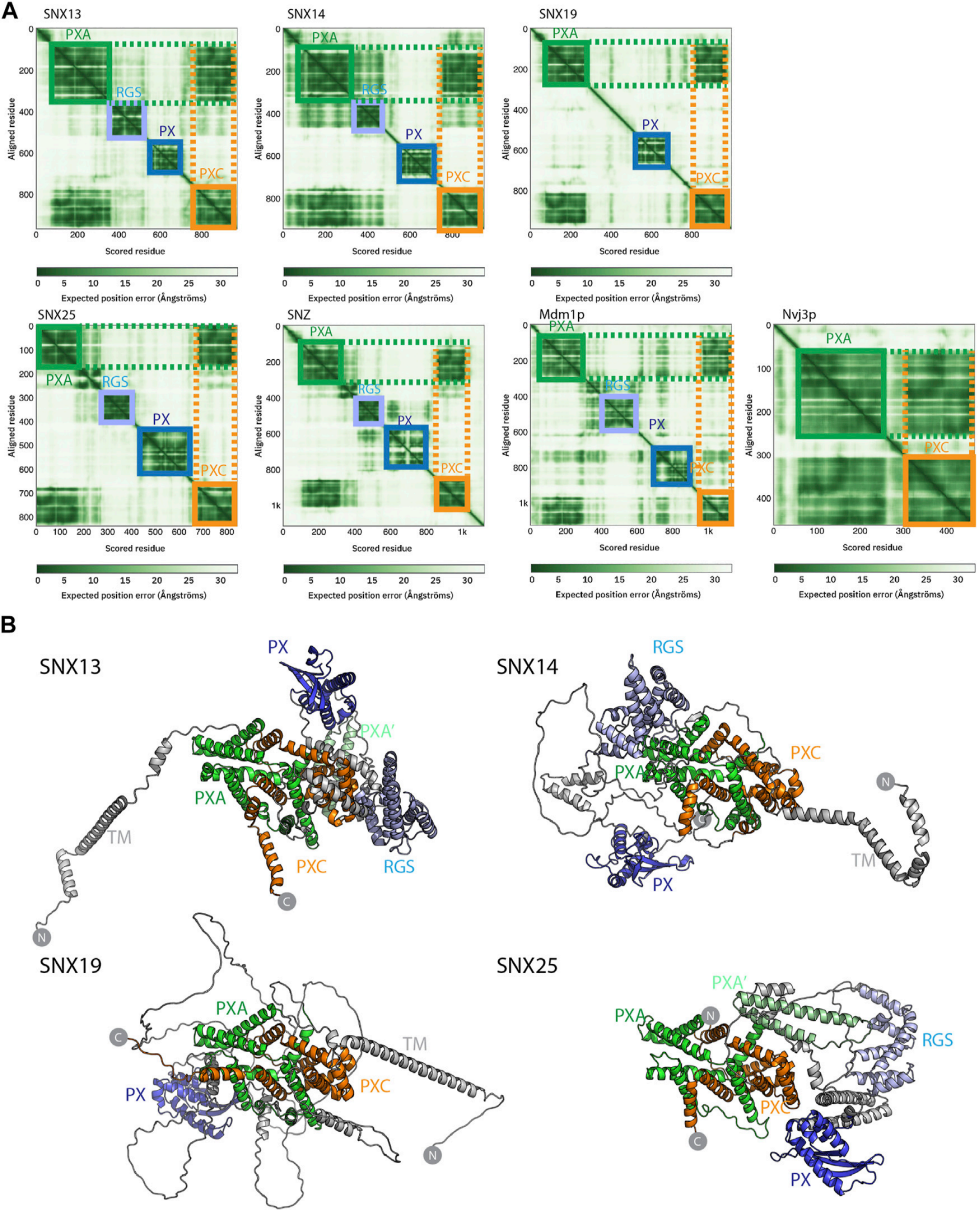
Structures of the SNX-RGS proteins predicted by AlphaFold2. **(A)** Predicted Alignment Error (PAE) plots from the AlphaFold2 database (Tunyasuvunakool et al., 2021) are shown for human fly and yeast SNX-RGS proteins. In these plots all SNX-RGS proteins show a strong degree of correlation between the PXA and PXC domain suggesting these two domains are physically associated. **(B)** The predicted structures of human SNX-RGS proteins from the AlphaFold2 database. The PXA domain is coloured green, RGS domain in light blue, PX domain in blue and PXC domain in orange. The predicted TM domain and any unstructured linker regions are coloured grey. The structures are shown in the same orientation after alignments based on the PXA and PXC core region. This shows that the two domains are intimately entwined with each other, whereas the TM, RGS and PX domains are predicted to have flexible orientations relative to these domains.

The human SNX25 open reading frame (ORF) cloned into pcDNA3.1+-C DYK was obtained from Genscript (NM_031953). This expresses a C-terminally tagged SNX25 (SNX25-FLAG) that has the same sequence as the AlphaFold2 model shown in Figure 2B. Note however, that it lacks the predicted N-terminal ER anchor found in the SNX25 isoform A0A494C0S0 that is modelled in Supplementary Figure S4. A431 cells were maintained in DMEM medium (Gibco Life technologies) supplemented with 10% foetal bovine serum (FBS) and Penicillin/Streptomycin. A431 cell line was sourced from ATCC and tested regularly for mycoplasma contamination. Cells (2 × 10^5^) were plated in six well culture dishes (Nunc^™^, Cat. No. 140675, Culture area—9.6 cm^2^) and grown to 50% confluency before transfection of SNX25-FLAG construct using lipofectamine 3000 (Invitrogen) as per manufacturer’s protocol. Cells were shifted to DMEM media containing oleic acid (50 µg/ml) 4 h post transfection and incubated for another 20 h. DMEM with oleic acid was prepared by mixing oleic acid (Sigma Aldrich, Cat. No. O-1008) in DMEM medium containing bovine serum albumin (fatty acid free) to final oleic acid concentration (50 µg/ml) with rigorous shaking at 37°C to avoid precipitation. Cells were fixed 24 h post-transfection with 4% paraformaldehyde in phosphate buffered saline (PBS) at 4°C and subsequently permeabilised with 0.1% Triton X-100 in PBS for 7 min. Cells were probed with FLAG antibody (Sigma Aldrich Cat. No. F7425, 2 µg/ml) and anti-Rabbit secondary antibody Alexa Fluor^®^488 conjugate (4 µg/ml) to visualise SNX 25 protein. Cells were finally labelled with nile red solution (100 nM) to visualise lipid droplets. Confocal images (1024 × 1024) were acquired on a Zeiss inverted LSM 880 microscope coupled with fast airyscan detector (Carl Zeiss, Inc) equipped with ×63 oil immersion objective, NA 1.4.

## Results

### Crystal Structure of the Human SNX25 RGS Domain

The SNX-RGS family members are typically represented as modular proteins with four to five distinct conserved domains (Figures 1A,B) (Amatya et al., 2021; Mas et al., 2014; Henne et al., 2015; Saric et al., 2021; Hariri et al., 2019; Akizu et al., 2015; Thomas et al., 2014; Suh et al., 2008; Zheng et al., 2001; Datta et al., 2020; Li et al., 2014). Our previous studies examined the structures and phosphoinositide binding properties of the PX domains of SNX14, SNX19 and SNX25 using NMR and X-ray crystallography (Chandra et al., 2019; Mas et al., 2014; Ugrankar et al., 2019). Here we have also solved the structure of the human SNX25 RGS domain by X-ray crystallography in two crystal forms (Figure 1C and Supplementary Figure S1 and Supplementary Table S1). The wildtype sequence of the RGS domain crystallises with a non-native disulfide bond formed by Cys526 between adjacent monomers in the crystal lattice which distorts the orientation of the helix α5. Mutation of Cys526 to alanine (C526A) prevents this bond formation and allows the domain to crystallise with a more typical α5 conformation similar for example to a canonical RGS domain such as RGS16 (PDB ID 2IK8) (Soundararajan et al., 2008) (Figure 1D).

We next investigated how similar the experimentally determined SNX25 RGS domain was to the predicted structure from the AlphaFold2 database (Tunyasuvunakool et al., 2021). The structure predicted by AlphaFold2 is remarkably close to the SNX25 crystal structure, with an overall root-mean-squared-deviation (RMSD) of 0.8 Å over 107 C_α_ atoms (Figure 1E). Side chain conformations in general were likewise closely aligned between the experimental and predicted structures (Supplementary Figure S2). The predicted structures of the human SNX13 and SNX14 RGS domains from the AlphaFold2 database are generally similar to the experimental SNX25 RGS domain structure as expected, but with some notable differences (Figure 1F). In general, the three domains have relatively low sequence homology, and this is reflected for example in a significantly longer α3-α4 extension in SNX13 compared to the other two proteins and (Figures 1F,G). The experimental structures of the PX domains of SNX14, SNX19 and SNX25 were also compared with their respective AlphaFold2 predictions and in general, the AlphaFold2 models were found to be highly similar with only minor differences in sidechain positions and in some flexible loop regions (Supplementary Figure S2). Overall, these comparisons confirm that the AlphaFold2 predictions of selected SNX-RGS domains are accurate models comparable to their known experimental structures, including the RGS domain of SNX25 for which no detailed structural information was available prior to this study.

### AlphaFold2 Predictions of the Yeast, Fly and Human SNX-RGS Proteins Reveal Conserved PXA and PXC Domain Intramolecular Interactions

Despite these structural insights into the SNX-RGS proteins, the N-terminal PXA and PXC domains bear no obvious sequence similarity to other proteins and their structures have not been determined, and the overall architecture of the proteins is also unknown. We have so far been unsuccessful in expressing and purifying either full-length SNX-RGS proteins or any of their individual PXA or PXC domains in suitable quantities for structural or biochemical analyses. As the recently released AlphaFold2 database (Tunyasuvunakool et al., 2021) includes predictions of the proteomes of multiple eukaryotic species from yeast to humans, we took advantage of this to examine the predicted structures of full-length SNX-RGS proteins across different species (Figure 2 and Supplementary Figures S2, S3).

The plots of the predicted alignment errors (PAE) of the AlphaFold2 models provide important insights into the organisation of the full-length proteins (Figure 2A). The PAE measures AlphaFold’s expected position error at residue “x” if the predicted and true structures were aligned on residue “y.” Square regions of the plot along the diagonal indicate the presence of globular domains where residues are structurally correlated with each other, while off-diagonal low PAE regions for residue pairs from two different domains is highly indicative of stable inter-domain interactions. The PAE plots for the human SNX-RGS proteins display expected low PAE regions for each of the four cytoplasmic domains downstream of the N-terminal ER membrane anchor (except for SNX19 which lacks an RGS domain). Notably, even though the N-terminal PXA domain and C-terminal PXC domains are around 500 amino-acids apart in the primary sequence, these two domains are invariably predicted to be tightly correlated with each other. Further, *Drosophila* Snz and yeast Mdm1p homologues show a similar high degree of structural correlation between their PXA and PXC domains.

The AlphaFold2 predicted atomic structures of the human SNX-RGS proteins are shown in Figure 2B and Supplementary Figure S3. Consistent with secondary structure predictions (Mas et al., 2014) both the PXA and PXC domains are entirely α-helical, and as indicated by the PAE plots they are tightly associated with each other. Indeed, they form an overall structure with a highly entwined topology, which is structurally similar in all four proteins. Relative to this “core” structure the N-terminal membrane anchor, and intervening RGS and PX domains adopt diverse orientations pointing to a substantial degree of structural dynamics in the full-length proteins. Note that the human SNX25 sequence in the AlphaFold2 database (Uniprot ID Q9H3E2) lacks the N-terminal membrane anchor as previously identified (Mas et al., 2014; Lauzier et al., 2021) (Uniprot A0A494C0S0). We therefore also performed predictions of the full SNX25 sequence using the online ColabFold pipeline (Mirdita et al., 2021), which show a similar structure but with the additional N-terminal α-helical membrane anchor (Supplementary Figure S4). As for predictions across the family, the three separate models of human SNX25 generated by ColabFold suggest that the transmembrane anchor, RGS and PX domains adopt relatively flexible orientations with respect to the core PXA-PXC structure. Although not shown, both full-length Mdm1p and Snz have analogous architectures with a core structure composed of the PXA and PXC domains that adopt a flexible orientation with respect to the transmembrane, RGS and PX domains. Overall, the predicted structures of the RGS-SNX proteins are highly consistent across homologues and species and reveal a novel intramolecular association between the previously uncharacterised PXA and PXC domains.

### The PXA and PXC Domains Form an Interwoven Structure with a Large Conserved Hydrophobic Channel

**FIGURE 3:**
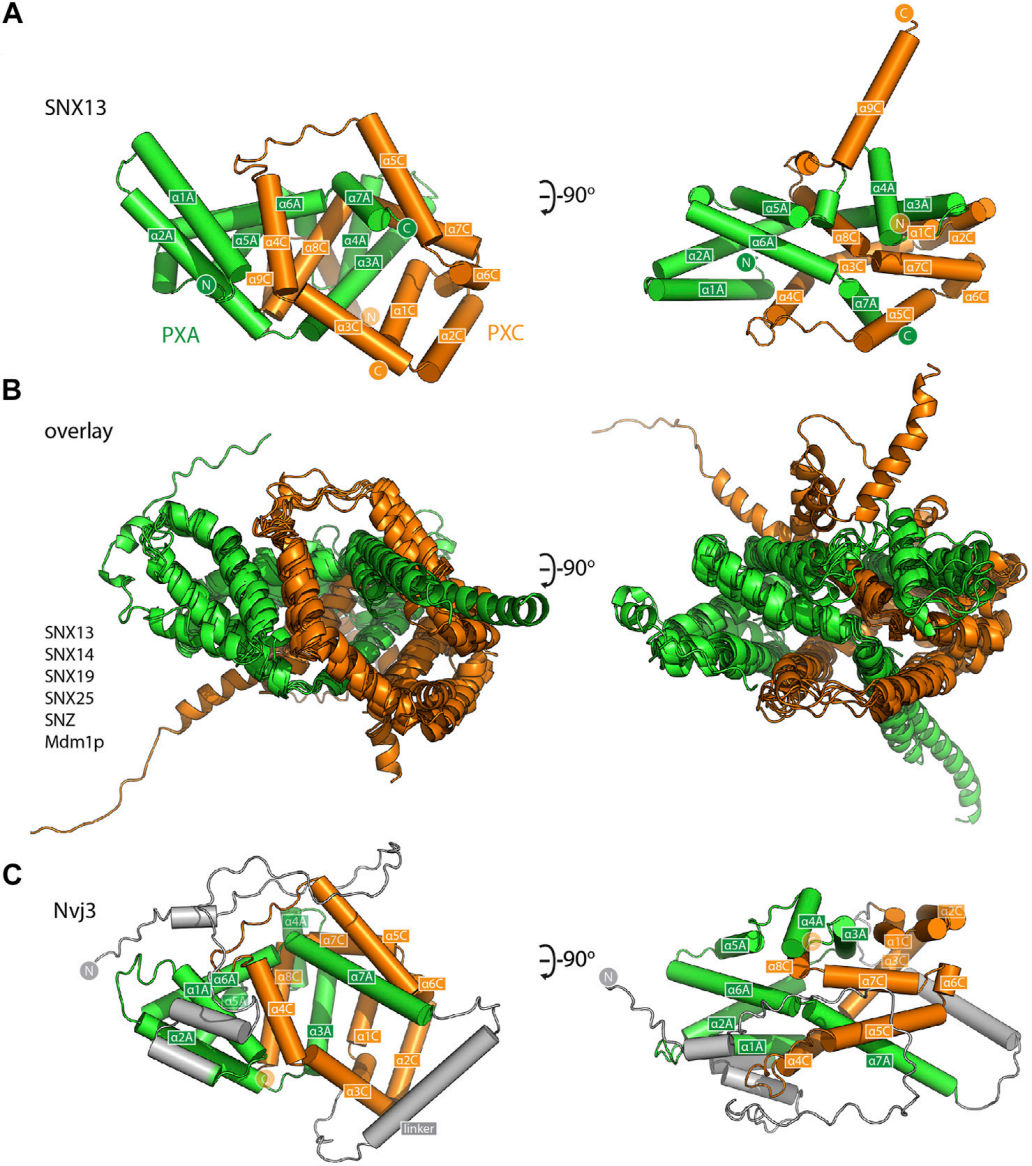
The PXA and PXC domains combine to form an intertwined α-helical structure. **(A)** Structure of the PXA and PXC domains of human SNX13 predicted by AlphaFold2 in green and orange respectively, shown with cylinders for α-helices. The two domains are predicted to be tightly interwoven. **(B)** An overlay of the core PXA-PXC domains of human, yeast and fly SNX-RGS proteins shows that all predicted structures have the same topology. **(C)** Predicted structure of *S. cerevisiae* Nvj3 with the regions expected to be similar to PXA and PXC domains coloured green and orange and the linker shown in grey.

For a closer examination of the PXA-PXC structure we focus on these domains from the SNX13 model (Figure 3A and Supplementary Movie S1). Both the PXA and PXC domains are entirely α-helical as predicted (Mas et al., 2014) and do not share any obvious structural similarity with previously characterised proteins. A notable feature of their structures is that they are intimately intertwined in such a way that neither domain would be expected to form their correct fold independently of the other. The threading of their α-helical structures together forms an overall elongated structure with a length of around 80 Å. The same α-helical topology and pattern of threading between the PXA and PXC domains is highly conserved and is seen in all SNX-RGS family members (Figure 3B). Although the yeast protein Nvj3p (Ydr179w-a) is not found in higher eukaryotes, it was previously proposed to share weak sequence similarity with the PXA domain of the SNX-RGS proteins (Henne et al., 2015). Interestingly the AlphaFold2 prediction of Nvj3p shows a very similar structure to the combined PXA and PXC fold of the SNX-RGS proteins (Figure 3C). Although it lacks a transmembrane, RGS or PX domains, and has only a minimal linker sequence between the two halves of its structure, it shares the same overall topology and is a bona fide PXA-PXC structure.

**FIGURE 4:**
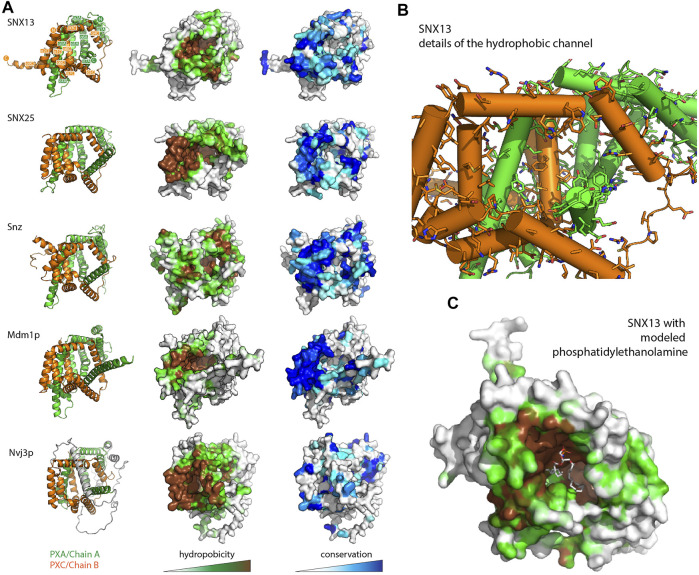
The PXA-PXC structure forms a conserved hydrophobic cavity with potential for lipid binding. **(A)** The predicted structures of PXA-PXC domains from human SNX13 and SNX25, fly Snz, and yeast Mdm1 and Nvj3 are shown in ribbon representation (left), surface coloured by hydrophobicity (middle), and sequence conservation (right). All PXA-PXC structures have a highly conserved hydrophobic tunnel. **(B)** Structure of PXA and PXC domains of SNX13 with sidechains of the putative lipid binding pocket shown. **(C)** The PXA and PXC domains of SNX13 is shown in close-up with its surface coloured for hydrophobicity as in **(A)**. A phosphatidylethanolamine lipid has been docked manually in the putative lipid binding pocket to give perspective on the dimensions of the channel.

A visual inspection of the core PXA-PXC domains reveals the presence of a large channel that runs through much of the structure with an exposed surface composed of side chains of both domains (Figure 4 and Supplementary Figure S5 and Supplementary Movie S1). The channels are extremely hydrophobic, as seen from mapping surface hydrophobicity (Figure 4A and Supplementary Figure S5) or from inspecting the details of the side chains lining the channel of human SNX13 as an example (Figure 4B and Supplementary Figure S5). The entrance to the cavities is highly conserved indicating it is likely to be a critical functional element of the proteins (Figure 4A and Supplementary Figure S5). The presence of such a hydrophobic channel or pocket is a common feature of the class of proteins known as lipid transfer proteins (LTPs) which can bind, extract and transport hydrophobic lipids and fatty acids from various compartments for non-vesicular trafficking (Malinina et al., 2017; Wong et al., 2017; Wong et al., 2019; Chiapparino et al., 2016) (Supplementary Figure S5). Given this structural similarity, coupled with the known localisation of the SNX-RGS proteins at ER membrane contact sites and their effects on lipid homeostasis, we propose that this channel in the SNX-RGS proteins is very likely to be a lipid binding pocket. The sizes of the cavities found in the various PXA-PXC proteins are relatively large, ranging from ∼2000–6000 Å^3^ compared with typical sized lipid binding pockets in other LTPs of ∼400–2000 Å^3^ (Supplementary Figure S5 and Supplementary Table S3). These cavities are larger than those found in cholesteryl ester transfer protein (CETP), which can bind up to two phospholipids and two neutral lipids such as cholesteryl esters or triglycerides at once (Supplementary Figure S5) (Qiu et al., 2007). Manual placement of a phosphatidylethanolamine lipid (volume ∼1200 Å^3^ for palmitoyl-oleoyl-phosphatidylethanolamine (Kučerka et al., 2015)) in the channel of SNX13 shows that the dimensions of this hydrophobic pocket in the SNX-RGS proteins is well suited for binding lipid acyl chains (Figure 4C and Supplementary Movie S1).

**FIGURE 5:**
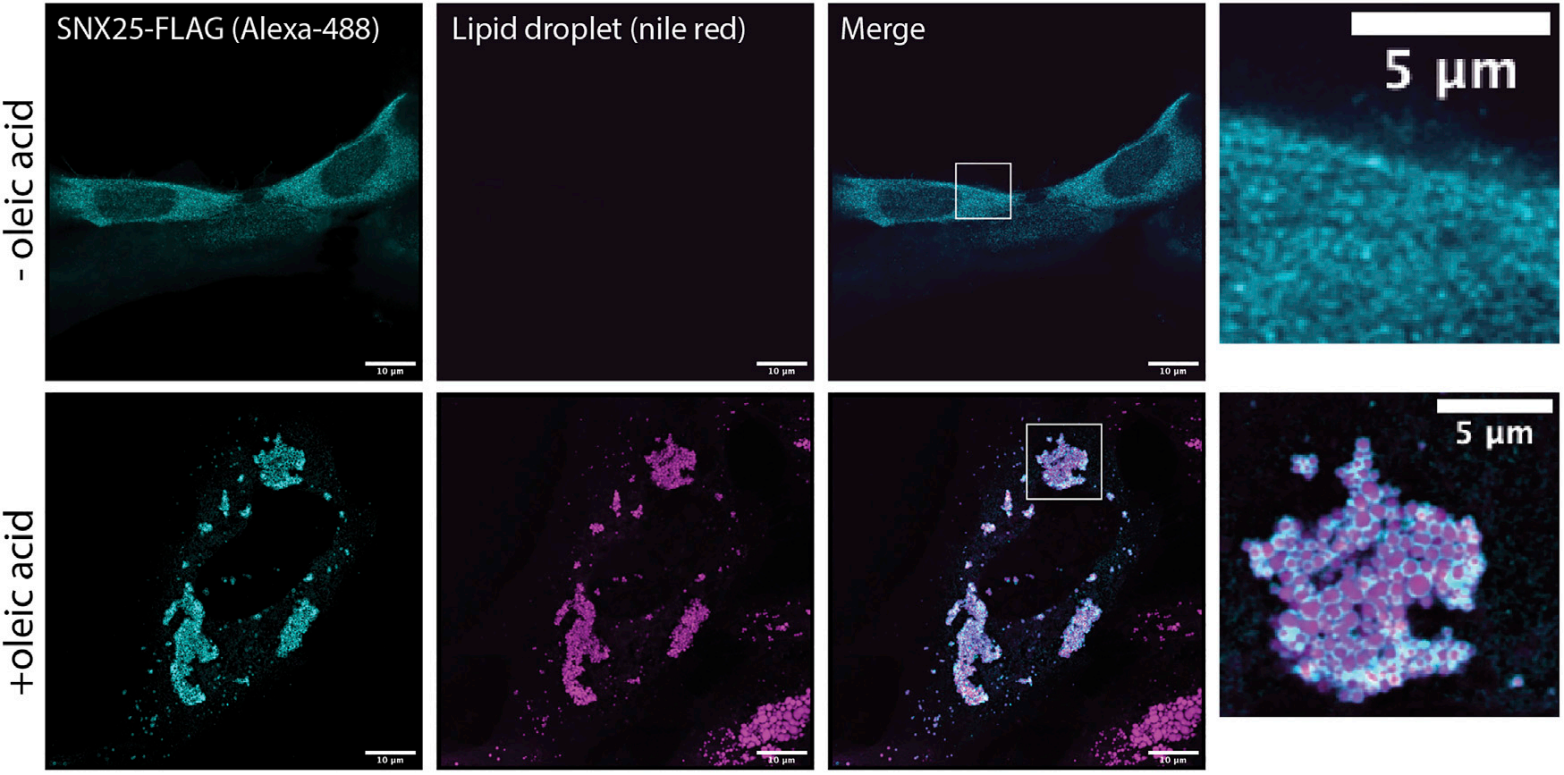
Human SNX25 is localises to newly synthesised LDs following oleic acid addition. A431 cells were transfected with SNX25-FLAG and either left untreated or treated with 50 μg/ml oleic acid to stimulate LD formation. After oleic acid addition SNX25-FLAG undergoes rapid redistribution to the periphery of newly generated LDs LD periphery. Scale bars represent 10 μm.

Several previous studies have shown that the SNX-RGS proteins are rapidly recruited to the surface of newly formed lipid droplets. This has been observed for Mdm1 in yeast (Hariri et al., 2018), Snz in flies (Ugrankar et al., 2019), and SNX13, SNX14, and SNX19 in mammalian cells (Bryant et al., 2018; Datta et al., 2019; Saric et al., 2021; Lu et al., 2022). This provides strong support for a role in direct transfer of neutral lipids, phospholipids, or lipid metabolites at ER-LD contact sites. We confirmed the ability to localise to the LD surface is conserved in all human family members by analysing the localisation of SNX25 (Figure 5). In A431 cells in the absence of stimulation SNX25 shows a relatively diffuse localisation, however on addition of oleic acid SNX25-FLAG is rapidly redistributed and shows a strong degree of localisation to the surface of newly formed LDs. Further work will be needed to examine if these ER-associated LDs are also in contact with other membrane compartments, but this data confirms that all SNX-RGS family members from yeast to humans have a strong affinity for the LD monolayer.

### Structural Bioinformatics of the SNX-RGS Proteins

The DALI webserver (Holm, 2020) is now capable of searching the entire database of AlphaFold2 predictions (Tunyasuvunakool et al., 2021) for structurally similar proteins. We performed a search using the core PXA-PXC structure of SNX25 to determine if any other proteins share similar predicted folds (Supplementary Table S2). This revealed several interesting observations. Firstly, while the PXA-PXC structure is found in proteins across metazoans, plants and yeast we did not find any similar structures in either *D. dictyostelium* or *P. falciparum* and they therefore do not seem to be present in all eukaryotes. Secondly, we identified three proteins in the fission yeast *S. pombe* with this fold. Meiotically up-regulated gene 122 protein (Mug122) (O74444) lacks the RGS domain but has both transmembrane and central PX domains, while snx12 (Q9USN1) has each of the domains common to the SNX-RGS family. The protein annotated as Pxa1 (O14200) appears to be most similar to Nvj3, as it lacks transmembrane, PX and RGS domains. We also performed a BLAST sequence search of the *Chaetomium thermophilum* genome, a thermophilic filamentous yeast popular with structural biologists because of the generally higher stability of the encoded proteins. The *C. thermophilum* genome encodes three proteins with putative PXA-PXC structures (Supplementary Table S2), and these were confirmed by performing ColabFold predictions of their structures (not shown). One of these has all domains common to the SNX-RGS family, a second lacks the RGS domain like human SNX19, and the third has only the PXA-PXC domains like *S. cerevisiae* Nvj3.

### Lec1/Ypr097w Is Another Putative LTP Found in Yeast and Ameoba

**FIGURE 6:**
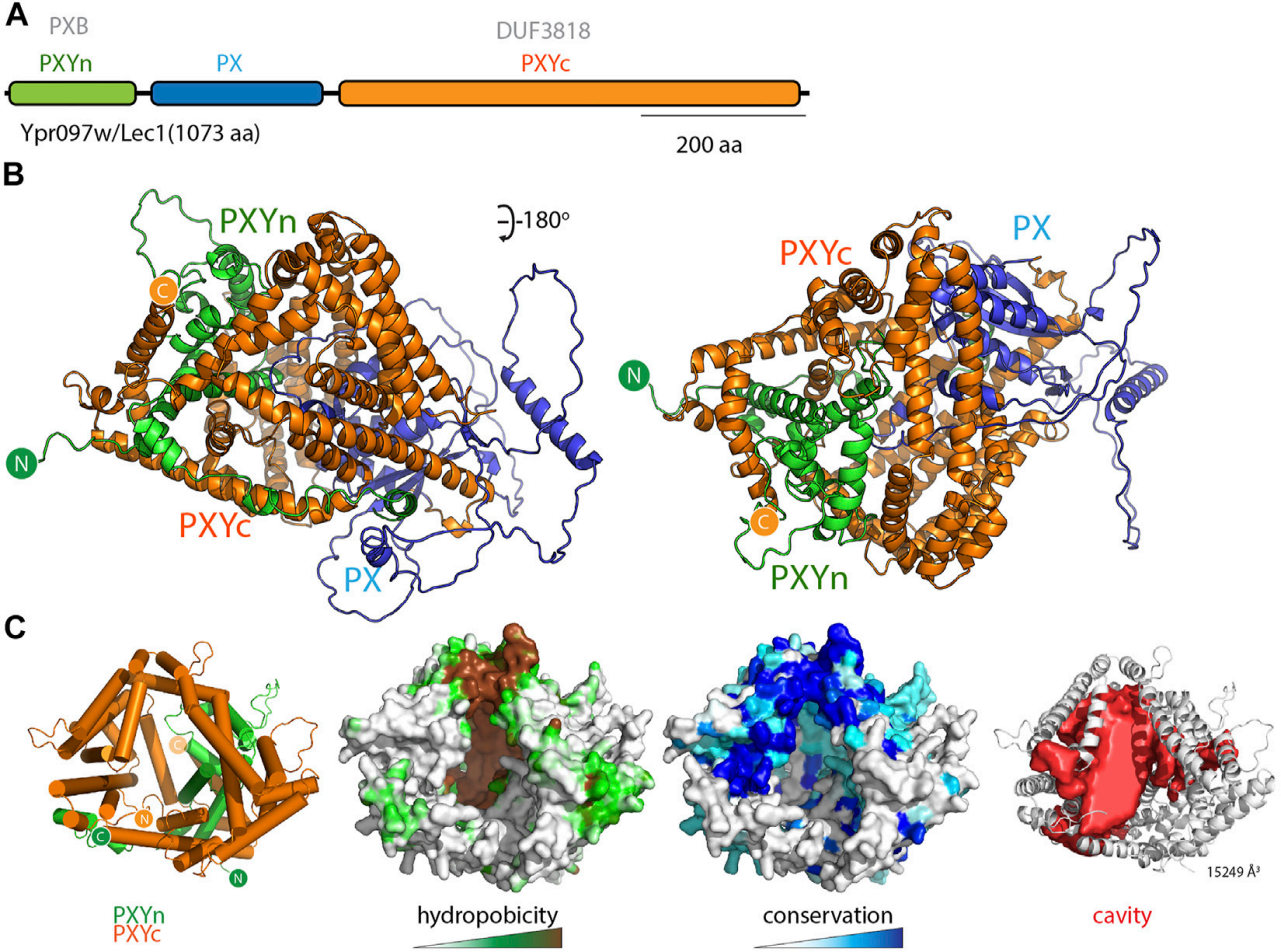
The yeast Lec1/Ypr097w protein forms a unique structure with a hydrophobic cavity. **(A)** Domain structure of the *S. cerevisiae* Lec1/Ypr097w protein. **(B)** AlphaFold2 structural prediction of the Lec1/Ypr097w protein with the PXYn domain in green, PX domain in blue and PXYc domain in orange. **(C)** The PXYn and PXYc domains are shown with α-helices in cylinder representation and the PX domain removed for clarity. The domains encompass a large conserved hydrophobic cavity and like the SNX-RGS proteins may be a potential lipid binding protein. The far right panel shows the solvent accessible cavity (red surface representation) identified with POCASA (Yu et al., 2010) and the volume (Å^3^) of the largest identified cavity is indicated (Roderick et al., 2002; Samygina et al., 2011; Schauder et al., 2014; Dong et al., 2019).

While examining the AlphaFold2 predicted structures of PX domain containing proteins in various species, and primed by the discovery of the PXA-PXC lipid-binding fold, we noticed that another yeast protein called Lec1/Ypr097w (Q06839) also possessed a superficially similar architecture. Lec1/Ypr097w is 1073 residues in length and is annotated to have a central PX domain between an N-terminal PXB domain and C-terminal domain of unknown function (DUF3818) (Figure 6A). Analogously to the SNX-RGS proteins, the N- and C-terminal domains of Lec1/Ypr097w form α-helical structures with previously unknown folds that come together to form an intimately interwoven structure (Figure 6B; Supplementary Figures S6A,B). Despite this superficial similarity however, neither domain is related in sequence or topology to the PXA or PXC domains and the way they interact with each other is also entirely different. Note that the term “PXB domain” is also used for a C-terminal domain found in human SNX20 and SNX21 which is completely unrelated (Clairfeuille et al., 2015). For clarity herein we will refer to the N-terminal Lec1/Ypr097w PXB domain as “PXYn” and the C-terminal DUF3818 domain as “PXYc,” where PXY denotes “PX-associated domains from yeast.”

Most interestingly, the PXYn and PXYc domains of Lec1/Ypr097w also form a barrel-like structure that has a very large cavity surrounded by hydrophobic residues (Figure 6C and Supplementary Table S3). The structure is conserved in homologues from *Schizosaccharomyces pombe* (Q9Y7N9), and *Candida albicans* (A0A1D8PQ52) (Supplementary Figure S6C). Although structurally unrelated to the SNX-RGS proteins, the general similarities to Lec1/Ypr097w and its homologues are striking, with a central phosphoinositide binding PX domain flanked by N-terminal and C-terminal α-helical domains that come together to form a co-folded intramolecular structure. Like the SNX-RGS proteins based on its structural properties it is very likely that the hydrophobic cavity formed by the PXYn-PXYc domains is also involved in binding to lipids or fatty acids. The volume of the cavity in Lec1/Ypr097w is very large (>15000 Å^3^) and could potentially accommodate multiple phospholipid or neutral lipid molecules. In each of the predicted PXY protein structures from different species the PX domain forms a loose plug over the large hydrophobic cavity suggesting a potential regulatory role for this domain in lipid binding (Supplementary Figure S6C). This may be relatively dynamic as several independent predictions of Lec1/Ypr097w using ColabFold suggest the PX domain can adopt alternative orientations that either block the pocket or leave the cavity exposed (Supplementary Movie S2).

The PXYn-PXYc structure appears to be relatively restricted throughout evolution, as we only found examples in yeast, including two proteins in *S. pombe*, as well as a protein in *D. dictyostelium. S. pombe* PX domain-containing protein C1450.12 (Q9Y7N9) is highly akin to Lec1/Ypr097w from *S. cerevisiae,* while uncharacterized protein C663.15c (O74521) appears to lack a central PX domain between the two halves of the PXYn-PXYc structure (Supplementary Figures S6, S7). The *D. discoideum* DUF3818 domain-containing protein (Q54JB8) also lacks a PX domain, and while its C-terminal PXYc domain is like Lec1/Ypr197w, its N-terminal region is structurally divergent from the PXYn domain (Supplementary Figure S7). Despite this it also possesses a predicted large hydrophobic cavity and therefore may share a functional relationship. Again, we performed a BLAST search of the *C. thermophilum* protein sequences using both *S. pombe* sequences as inputs and found one protein homologous to the Lec1/Ypr097w-like Q9Y7N9 (G0SCP5) and a protein lacking the PX domain like O74521 (G0S997). These discoveries further demonstrate the utility of AlphaFold2 for predicting novel structures, and its power in aiding evolutionary analyses of protein homology.

## Discussion

With the recent advances made by the AlphaFold2 machine learning algorithm and public release of the AlphaFold2 database (Jumper et al., 2021; Tunyasuvunakool et al., 2021), it is now possible to perform detailed structural analyses and evolutionary comparisons of protein families across diverse species. In this work we have solved the crystal structure of the human SNX25 RGS domain and gone on to examine the previously unexplored structures of the full-length SNX-RGS proteins. Although the overall structures of these proteins display conformational flexibility with respect to their N-terminal transmembrane ER tethers, in contrast to previous pictures based on primary and secondary structure predictions the disparate N- and C-terminal PXA and PXC domains form a highly entwined intramolecular interaction generating a compact cylindrical α-helical structure. The most notable feature of a central hydrophobic cavity with a highly conserved entrance and surface lining. This leads us to conclude that the likely function of the PXA-PXC structure will be to bind and transport lipids or fatty acids, and that the proteins represent a distinct class of ER membrane-anchored LTPs.

Broader analysis of other PX domain-containing protein family members indicate that the yeast protein Lec1/Ypr097w and related molecules share a superficially similar LTP structure and function. Here the central PX domain is found between N-terminal PXYn and C-terminal PXYc domains that also become conjoined to form an all α-helical barrel-like structure with a large hydrophobic cavity, although their sequences are not related and their structural details are entirely different. As we were performing these studies, a preprint was published by the Schuldiner lab that identified novel proteins that reside at or regulate formation of membrane contact sites in yeast (Castro et al., 2021). In this work Ypr097w was identified to localise to contacts between lipid droplets and the cell surface and renamed Lec1 (Lipid Droplet Ergosterol Cortex 1) because of a potential role in ergosterol transport. This team also analysed the AlphaFold2 predicted structure of Lec1/Ypr097w and came to similar conclusions about its potential role as a novel lipid transfer protein (LTP), as well as identifying the superficial structural similarities to the SNX-RGS proteins.

What do these new structural insights suggest about the function(s) of the SNX-RGS proteins? The hydrophobic nature and conserved surface of the large interior cavity found in the PXA-PXC domain structure indicates they are very likely to mediate the binding of lipids, fatty acids or other lipid-derived metabolites. A general finding is that the SNX-RGS proteins reside in the ER at steady state *via* their N-terminal membrane anchor, and subsequently colocalise with LDs particularly under conditions of new LD synthesis. They are typically found to be enriched in regions of the ER and ER-associated LDs that are in contact with other membranes where they can play a tethering role *via* their phosphoinositide-binding PX domain. These membrane contacts include the cell surface in the fat body cells of fruit flies (Ugrankar et al., 2019), the nuclear-vacuolar junction in budding yeast (Henne et al., 2015; Hariri et al., 2018; Hariri et al., 2019), and with endolysosomal compartments in mammalian cells (Bryant et al., 2018; Datta et al., 2019; Saric et al., 2021; Lu et al., 2022). This localisation to ER-LD-membrane contact sites is dramatically altered under conditions of lipid flux (e.g. addition of excess free fatty acids to cells (Datta et al., 2019; Saric et al., 2021)), and the SNX-RGS proteins play important roles in lipid metabolism and homeostasis.

The effects of mutations, knockouts or overexpression of SNX-RGS proteins are complex, but at the cellular level their depletion commonly leads to higher cellular levels of neutral triacylglycerol (TAG) and accumulation of cholesterol in late endosomal compartments (Bryant et al., 2018; Hariri et al., 2018; Bryant et al., 2020; Lu et al., 2022). Yeast Mdm1 and human SNX14 have been associated with Faa1 and ACSL3 respectively, long-chain-fatty-acid--CoA ligases that activate fatty acids for synthesis of cellular lipids and degradation *via* β-oxidation (Hariri et al., 2018; Datta et al., 2019). Drosophila Snz and human SNX14 were also found to associate with stearoyl-CoA desaturases Desat1 and SCD1 respectively, which catalyze the insertion of a cis double bond at the Δ-9 position of fatty acyl-CoA substrates (Ugrankar et al., 2019; Datta et al., 2020). Knockout of SNX14 causes cells to be sensitive to saturated fatty acid (SFA)-induced toxicity and increased levels of SFAs are incorporated into phospholipids, which can be rescued by SCD1 overexpression. A recent CRISPR screen for genes that influence cholesterol homeostasis found SNX13 (and SNX14) as a prominent hit (Lu et al., 2022). Niemann Pick type C (NPC) disease is caused by genetic defects in the lysosomal cholesterol transport system, Niemann Pick C1 and C2 proteins (NPC1 and NPC2) leading to endolysosomal accumulation of cholesterol and Bis(monoacylglycero)phosphate (BMP, also known as LBPA) (PentchevNiemann-Pick, 2004; Wheeler and Sillence, 2020), which is essential for normal cholesterol transport out of the endolysosomal compartment (Kobayashi et al., 1999; Chevallier et al., 2008; Gallala and Sandhoff, 2011; McCauliff et al., 2019; Gruenberg, 2020). In screens using the NPC1 inhibitor U18666A cholesterol accumulation could be reversed by depletion of SNX13, and cholesterol was exported in an NPC1-independent manner to other compartments including the plasma membrane. Surprisingly SNX13 depletion led to a significant increase in total lysosomal BMP, which may be important for the observed NPC1-independent cholesterol transport (Moreau et al., 2019; Ilnytska et al., 2021). Although highly speculative one possibility is that the PXA-PXC domains are important for transport and regulation of lipids such as BMP at contacts between the ER and endolysosomal compartments. Identifying specific ligand(s) and whether the different family members have alternative binding activities will require further experimentation.

The PXA-PXC domains were previously considered as conserved but essentially independent structures in the SNX-RGS family of proteins. From the analyses presented here it is clear that these two domains are intimately associated in a single structural unit and that neither the PXA or PXC sequences are likely to fold correctly in the absence of the other. Previously, several studies have examined the impact of deleting these regions on SNX-RGS protein function. While deletion of either domain generally renders the proteins non-functional as expected, there is evidence that the two sequences contain regions that are important for LD targeting and potentially protein interactions. In yeast, it was found that the PXA domain of Mdm1 could define sites of LD formation, and could recruit the Faa1 fatty-acid—CoA ligase (Hariri et al., 2019). In contrast, several reports have shown that the PXC domain by itself is required and sufficient for targeting to the surface of newly formed LDs in both flies and mammals (Datta et al., 2019; Ugrankar et al., 2019; Lu et al., 2022). Deleting an amphipathic helical region in SNX14 PXC (residues 801–819), which is predicted to sit at the entrance of the hydrophobic cavity of the assembled PXA-PXC structure (Supplementary Figure S5), prevents LD attachment. These studies suggest that even in the absence of the PXA domain, and thus proper folding of the PXA-PXC structure, there are sequences in the PXC domain capable of direct association with the LD monolayer.

In conclusion, although further experiments are required to experimentally validate these models and define the functional lipid interactions of the SNX-RGS proteins and their distantly related Lec1/Ypr097w cousins, these structural studies provide strong evidence for a novel lipid transport activity mediated by these conserved molecules. As a final general observation, this work further confirms the ability of AlphaFold2 to predict not only already known folds, but to define completely new structures without previously recognised topologies and thus provide new insights into biological function.

## Data Availability

The datasets presented in this study can be found in online repositories. The names of the repository/repositories and accession number(s) can be found below: http://www.wwpdb.org/, 7SR1, http://www.wwpdb.org/, 7SR2.
