# Supplementary material for: Structural Predictions of the SNX-RGS Proteins Suggest They Belong to a New Class of Lipid Transfer Proteins
Source: Front Cell Dev Biol. 2022 Feb 3;10:826688. doi: 10.3389/fcell.2022.826688 (PMC8864675; doi:10.3389/fcell.2022.826688)
Supplement: Supplementary file 2 [file Datasheet1.pdf]

Supplementary Information for

Structural predictions of the SNX-RGS proteins suggest they belong to a new class of lipid transfer proteins

Blessy Paul, Saroja Weeratunga, Vikas A. Tillu, Hanaa Hariri, W. Mike Henne and Brett M. Collins

b.collins@imb.uq.edu.au

Supplementary Figures

Figure S1. Crystal structure of the SNX25 RGS domain.

(**A**) Crystal structure of the wild-type human SNX25 RGS domain. (**B**) Alignment of the two chains in the asymmetric unit of wild-type SNX25 RGS domain. (**C**) The position of the non-native disulfide bond formed between adjacent chains in the crystal lattice. This causes the change in orientation of the α5 helix. (**D**) Crystal structure of the SNX25(C526A) mutant protein. (**E**) Alignment of the two chains in the asymmetric unit of SNX25(C526A) RGS domain. (**F**) Structural alignment of the wild-type and C526A SNX25 RGS domain structures.

Figure S2. Comparison of experimental SNX-RGS structures with their AlphaFold2 predictions.

(**A**) Crystal structure of the human SNX25(C326A) RGS domain (red, this study) aligned with the AlphaFold2 prediction (light blue). (**B**) Crystal structures of human SNX14 PX domain (green, PDB 4PQP; magenta, 4PQO) with the AlphaFold2 prediction (blue). (**C**) Crystal structure of mouse SNX19 PX domain (pink, PDB 4P2I) aligned with the AlphaFold2 prediction (blue). (**D**) NMR structure of human SNX25 PX domain (cyan, PDB 4PQP) aligned with the AlphaFold2 prediction (blue).

Figure S3. Additional details of the SNX13 predicted full length structure.

(**A**) AlphaFold2 prediction of full-length human SNX13 protein with individual domains coloured as in **Fig. 2B** with the TM domain and linker regions (grey), PXA domain (green), RGS domain (light blue), PX domain (blue), and PXC domain (orange). (**B**) AlphaFold2 prediction of full-length human SNX13 protein coloured according to the pLDDT score. (**C**) The AlphaFold2 predictions of human, mouse and zebrafish SNX13 proteins were aligned based on the core PXA-PXC structure.

Figure S4. AlphaFold2 prediction of full-length human SNX25 protein.

(**A**) Top ranked structure of full length human SNX25 [1] (Uniprot ID A0A494C0S0) predicted by ColabFold coloured according to pLDDT score. (**B**) Top ranked structure of human SNX25 predicted by ColabFold coloured according to the indicated domains. (**C**) Alignment based on the core PXA-PXC structure of the five structural predictions of human SNX25 from ColabFold.

Figure S5. Conserved hydrophobic cavities in all human, yeast and fly PXA-PXC domains.

Structures of indicated PXA-PXC domains in ribbon (left) surface coloured by hydrophobicity, and sequence conservation. A region in SNX14 is highlighted that when deleted prevents LD recruitment [2]. Far right panels display accessible cavities (red surface representation) in the proteins identified with POCASA[3] and the volume (Å^3) of the largest continuous cavity is indicated. The five bottom structures are examples of other lipid transfer proteins bound to different lipids, demonstrating how similar kinds of conserved hydrophobic pockets can serve as lipid binding cavities and provided for comparison. Extended synaptotagmin 2 (E-Syt2) is in complex with a phosphatidylethanolamine lipid

and Triton-X100 detergent molecule (PDB 4P42) [4]. Cholesteryl ester transfer protein (CETP) is in complex with two cholesteryl esters and two phosphatidylcholine lipids (PDB 2OBP) [5]. Glycolipid transfer protein (GLTP) is in complex with N-oleoyl-glucosylceramide (PDB 3S0K) [6]. Phosphatidylcholine transfer protein (PC-TP) is shown in complex with a phosphatidylcholine molecule (PDB 1LN2) [7]. Human OSBP-related protein 1 (ORP1) is shown bound to phosphatidylinositol(4,5)P_2 (PDB 5ZM6)[8]

Figure S6. AlphaFold2 predicted structure of Lec1/Ypr097w.

(**A**) Plot of the Predicted Alignment Error (PAE) from the AlphaFold2 database. There is a strong degree of correlation between the N-terminal PXYn and C-terminal PXYc domains suggesting these two domains are physically associated. (**B**) The predicted structure of Lec1/Ypr097w from S. cerevisiae coloured according to the pLDDT score. (**C**) Overlay of the PXY proteins from S. cerevisiae, S. pombe and C. albicans.

Figure S7. AlphaFold2 predicted structure of PXY domain proteins found in *S. pombe*, *C. thermophilum* and *D. dictyostelium*.

(**A**) Structural prediction of *S. pombe* Lec1/Ypr092w orthologue C1450.12. (**B**) Structural prediction of *S. pombe* Lec1/Ypr092w homologue C663.15c. (**C**) Structural prediction of *C. thermophilum* Lec1/Ypr092w orthologue EGS19171. (**D**) Structural prediction of *C. thermophilum* Lec1/Ypr092w homologue EGS20008. (**E**) Structural prediction of *D. discoideum* DUF3818 domain-containing protein.

Table S1. Statistics for X-ray crystallographic data collection and structure refinement[a]

	Human SNX25 RGS domain	Human SNX25 RGS domain (C526A)
**Wavelength (Å)**	1.006	0.95364
**Resolution range (Å)**	45.84 -2.4 (2.486-2.4)	48.25-2.42 (2.52-2.42)
**Space group**	P 1 21 1	P 1 21 1
**Unit cell**	48.6 57.1 67.0 90 109.5 90	44.0 75.9 51.1 90.00 109.3 90.00
**Total reflections**	47931 (3290)	81322 (7264)
**Unique reflections**	13200 (1003)	11898 (1111)
**Multiplicity**	3.6 (3.1)	6.8 (6.5)
**Completeness (%)**	96.5 (74.1)	97.9 (89.2)
**Mean I/sigma(I)**	11.9 (2.1)	7.2 (1.4)
**R-merge**	0.065 (0.506)	0.171 (0.976)
**R-meas**	0.089 (0.690)	0.185 (1.062)
**R-pim**	0.061 (0.467)	0.070 (0.410)
**CC1/2**	0.998 (0.718)	0.992 (0.631)
**R-work**	0.2164 (0.2984)	0.2108 (0.3258)
**R-free**	0.2421 (0.2906)	0.2497 (0.4051)
**Number of non-hydrogen atoms**	2157	2026
**macromolecules**	2080	1991
**solvent**	77	30
**RMS(bonds)**	0.003	0.007
**RMS(angles)**	0.55	0.87
**Ramachandran favored (%)**	97.08	97.38
**Ramachandran allowed (%)**	2.92	2.62
**Ramachandran outliers (%)**	0.00	0.00
**Clashscore**	2.66	6.07
**Average B-factor**	51.51	52.68
**macromolecules**	51.69	52.76
**solvent**	46.74	46.51
**PDB ID**	7SR1	7SR2

a. Statistics for the highest-resolution shell are shown in parentheses.

Table S2. Homologues of the PXA-PXC and PXYn-PXYc domain-containing proteins based on structural similarity in the AlphaFold2 database[a].

	PXA-PXC domains	Uniprot ID	PXYn-PXYc domains	Uniprot ID
H. sapiens **(human)**	SNX13<br>SNX14<br>SNX19<br>SNX25<br>SNX25 full length	Q9Y5W8<br>Q9Y5W7<br>Q92543<br>Q9H3E2<br>A0A494C0S0	-	-
D. melanogaster **(fly)**	Snz	Q9W3N0	-	-
C. elegans **(worm)**	snx-14<br>snx-13	G5EF63<br>Q9U2U6	-	-
A. thaliana **(plant)**	At1g15240<br>At2g15900	F4HZJ6<br>F4IJE1	-	-
D. discoideum **(amoeba)**	-	-	DDB0187809	Q54JB8
S. cerevisiae **(budding yeast)**	Mdm1/Yml104c<br>Nvj3/Ydr179W-a	Q01846<br>Q03983	Lec1/Ypr097w	Q06839
S. pombe **(fission yeast)**	snx12<br>mug122<br>pxa1	Q9USN1 (all domains)<br>O74444 (no RGS)<br>O14200 (like Nvj3)	SPCC663.15c<br>SPCC1450.12	O74521 (no PX)<br>Q9Y7N9 (all domains)
***C. thermophilum*[b]** **(thermophilic fungi)**	CTHT_0043790/EGS19887<br>CTHT_0051170/EGS18515<br>CTHT_0024100/EGS20576	G0S8X6 (all domains)<br>G0SDB4 (no RGS)<br>G0S5A3 (like Nvj3)	CTHT_0045050/EGS20008<br>CTHT_0057960/EGS19171	G0S997 (no PX)<br>G0SCP5 (all domains)
M. janaschii **(archaea)**	-	-	-	-
P. falciparum **(protozoan)**	-	-	-	-

a. The DALI webserver (http://ekhidna2.biocenter.helsinki.fi/dali/) was used to search the AlphaFold2 database against the indicates species using either the SNX25 PXA-PXC structure or the Lec1/Ypr097w PXYn-PXYc structure.
b. *C. thermophilum* proteins were identified via BLAST sequence searches and their domain structures were validated using ColabFold structural predictions.

Table S3. Cavity volumes identified in LTP proteins

**Protein**	**Largest continuous cavity volume ($Å^3$)[a]**
SNX13	2360
SNX14	2347
SNX19	3291
SNX25	3974
Snz	3830
Mdm1	2166
Nvj3	6166
Lec1/Ypr097w	15249
E-Syt2	1952
GLTP	380
PC-TP START domain	830
CETP	1873
ORP1	425

a. The cavity volumes in each structure were identified and calculated using POCASA (http://g6altair.sci.hokudai.ac.jp/g6/service/pocasa/)[3].

Table S4. Key Resources Table.

REAGENT or RESOURCE	SOURCE or REFERENCE	IDENTIFIER
**Bacterial Strains**		
E. coli DH5α	Invitrogen	18265017
BL21-CodonPlus (DE3)-RIL	Agilent Technologies	230245
**Mammalian cell Lines**		
A431 (human squamous carcinoma cell line)	Merck (ATCC)	85090402
**Antibodies, mammalian cell culture and imaging**		
FLAG	Sigma Aldrich	F7425
Nile red	Sigma Aldrich	72485
Donkey anti-Rabbit secondary antibody Alexa Fluor®488 conjugate	Life Technologies Australia	A21206
Culture dishes	Nunc	140675
DMEM, high glucose, pyruvate, no glutamine	Thermo Fisher Scientific Aus Pty Ltd	10313039
Foetal bovine serum	Cytiva (Global Life Sciences Solutions)	SH30084.03
Trypsin-EDTA 0.05%	Thermo Fisher Scientific Aus Pty Ltd	25300054
lipofectamine 3000	Thermo Fisher Scientific Aus Pty Ltd	L3000015
Penicillin/Streptomycin	Thermo Fisher Scientific Aus Pty Ltd	15070063
**Chemicals and enzymes**		
Thrombin	Sigma Aldrich	T6634-1KU
**Recombinant DNA**		
pGEX4T-2 human SNX25 RGS domain (residues 446-569; A0A494C0S0)	This study	N/A
pGEX4T-2 human SNX25 RGS domain (residues 446-569; A0A494C0S0) C526A mutation	This study	N/A
pcDNA3.1+-C DYK (FLAG) human SNX25	Genscript	NM_031953
**Deposited Data**		
human SNX13 AlphaFold2 model	[9, 10]	Q9Y5W8
human SNX14 AlphaFold2 model	[9, 10]	Q9Y5W7
human SNX19 AlphaFold2 model	[9, 10]	Q92543
human SNX25 AlphaFold2 model	[9, 10]	Q9H3E2
human SNX25 sequence including an N-terminal hydrophobic ER anchor sequence.	Uniprot	
fly Snz AlphaFold2 model	[9, 10]	Q9W3N0
yeast Mdm1 AlphaFold2 model	[9, 10]	Q01846
yeast Nvj3 AlphaFold2 model	[9, 10]	Q03983
yeast Lec1/Ypr097w AlphaFold2 model	[9, 10]	Q06839
S. pombe SNX14 AlphaFold2 model	[9, 10]	Q9Y7N9

C. albicans SNX14 AlphaFold2 model	[9, 10]	A0A1D8PQ52
human SNX14 PX domain crystal structure	[1]	4PQP
human SNX14 PX domain crystal structure	[1]	4PQO
mouse SNX19 PX domain crystal structure	[1]	4P2I
human SNX25 PX domain NMR structure	[11]	4PQP
human GLTP crystal structure	[6]	3S0K
human E-Syt2 crystal structure	[4]	4P42
Human phosphatidylcholine transfer protein (PC-TP) bound to phosphatidylcholine	[7]	1LN2
Human CETP bound to two cholesteryl esters and two phosphatidylcholine lipids	[5]	2OBD
Human OSBP-related protein 1 (ORP1) bound to phosphatidylinositol(4,5)P_2	[8]	5ZM6
human SNX25 RGS domain (residues 283-405; Q9H3E2) crystal structure	This study	7SR1
human SNX25 RGS domain (residues 283-405; Q9H3E2) (C326A) crystal structure	This study	7SR2
human RGS1 RGS domain crystal structure	[12]	2BV1
**Software**		
Coot	[13]	https://www2.mrc-lmb.cam.ac.uk/personal/pemsley/coot/
Pymol	Schrodinger, USA.	https://pymol.org/2/
AlphaFold2	[9]	N/A
ColabFold	[14]	https://colab.research.google.com/github/sokrypton/ColabFold/blob/main/beta/AlphaFold2_advanced.ipynb
Consurf	[15]	https://consurf.tau.ac.il
POCASA	[3]	http://g6altair.sci.hokudai.ac.jp/g6/service/pocasa/
**Other**		
HiLoad™ Superdex75 PG	GE Healthcare	Catalogue: 28989333

Supplementary References

1. Mas, C. *et al.* Structural basis for different phosphoinositide specificities of the PX domains of sorting nexins regulating G-protein signaling. *J Biol Chem* **289**, 28554-28568 (2014).

2. Datta, S., Liu, Y., Hariri, H., Bowerman, J. & Henne, W.M. Cerebellar ataxia disease-associated Snx14 promotes lipid droplet growth at ER-droplet contacts. *J Cell Biol* **218**, 1335-1351 (2019).

3. Yu, J., Zhou, Y., Tanaka, I. & Yao, M. Roll: a new algorithm for the detection of protein pockets and cavities with a rolling probe sphere. *Bioinformatics* **26**, 46-52 (2010).

4. Schauder, C.M. *et al.* Structure of a lipid-bound extended synaptotagmin indicates a role in lipid transfer. *Nature* **510**, 552-555 (2014).

5. Qiu, X. *et al.* Crystal structure of cholesteryl ester transfer protein reveals a long tunnel and four bound lipid molecules. *Nat Struct Mol Biol* **14**, 106-113 (2007).

6. Samygina, V.R. *et al.* Enhanced selectivity for sulfatide by engineered human glycolipid transfer protein. *Structure* **19**, 1644-1654 (2011).

7. Roderick, S.L. *et al.* Structure of human phosphatidylcholine transfer protein in complex with its ligand. *Nat Struct Biol* **9**, 507-511 (2002).

8. Dong, J. *et al.* Allosteric enhancement of ORP1-mediated cholesterol transport by PI(4,5)P2/PI(3,4)P2. *Nat Commun* **10**, 829 (2019).

9. Jumper, J. *et al.* Highly accurate protein structure prediction with AlphaFold. *Nature* **596**, 583-589 (2021).

10. Tunyasuvunakool, K. *et al.* Highly accurate protein structure prediction for the human proteome. *Nature* **596**, 590-596 (2021).

11. Chandra, M. *et al.* Classification of the human phox homology (PX) domains based on their phosphoinositide binding specificities. *Nat Commun* **10**, 1528 (2019).

12. Soundararajan, M. *et al.* Structural diversity in the RGS domain and its interaction with heterotrimeric G protein alpha-subunits. *Proc Natl Acad Sci U S A* **105**, 6457-6462 (2008).

13. Emsley, P. & Cowtan, K. Coot: model-building tools for molecular graphics. *Acta Crystallogr D Biol Crystallogr* **60**, 2126-2132 (2004).

14. Mirdita, M., Ovchinnikov, S. & Steinegger, M. ColabFold - Making protein folding accessible to all. . *bioRxiv* (2021).

15. Ashkenazy, H. *et al.* ConSurf 2016: an improved methodology to estimate and visualize evolutionary conservation in macromolecules. *Nucleic Acids Res* **44**, W344-350 (2016).

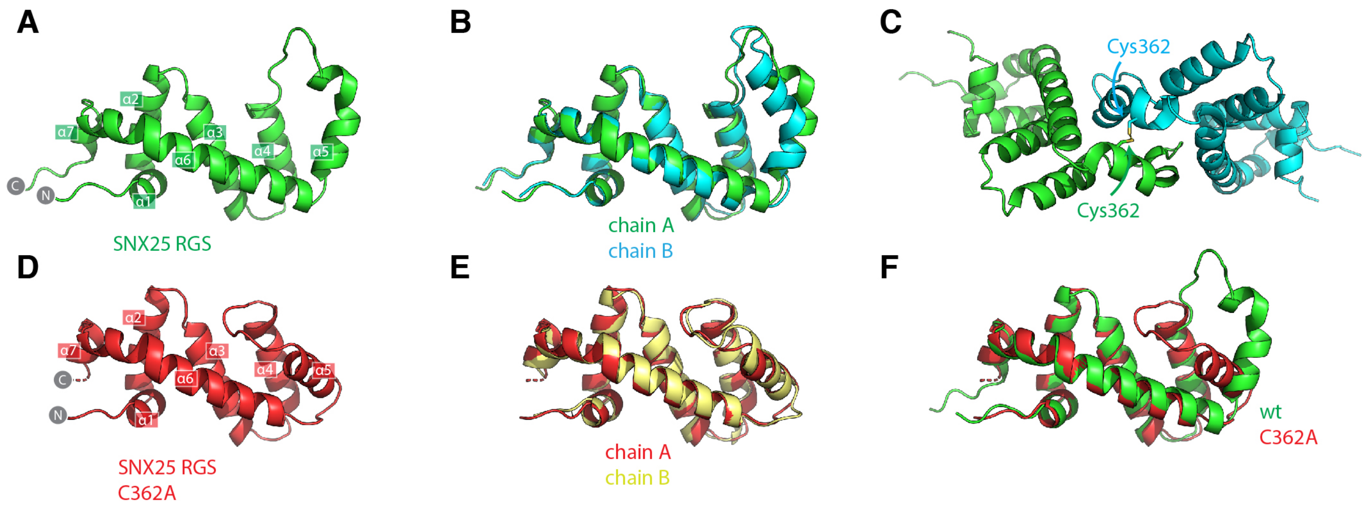

Figure S1. Crystal structure of the SNX25 RGS domain.
(A) Crystal structure of the wild-type human SNX25 RGS domain. (B) Alignment of the two chains in the asymmetric unit of wild-type SNX25 RGS domain. (C) The position of the non-native disulfide bond formed between adjacent chains in the crystal lattice. This causes the change in orientation of the [illegible]5 helix. (D) Crystal structure of the SNX25(C526A) mutant protein. (E) Alignment of the two chains in the asymmetric unit of SNX25(C526A) RGS domain. (F) Structural alignment of the wild-type and C526A SNX25 RGS domain structures.

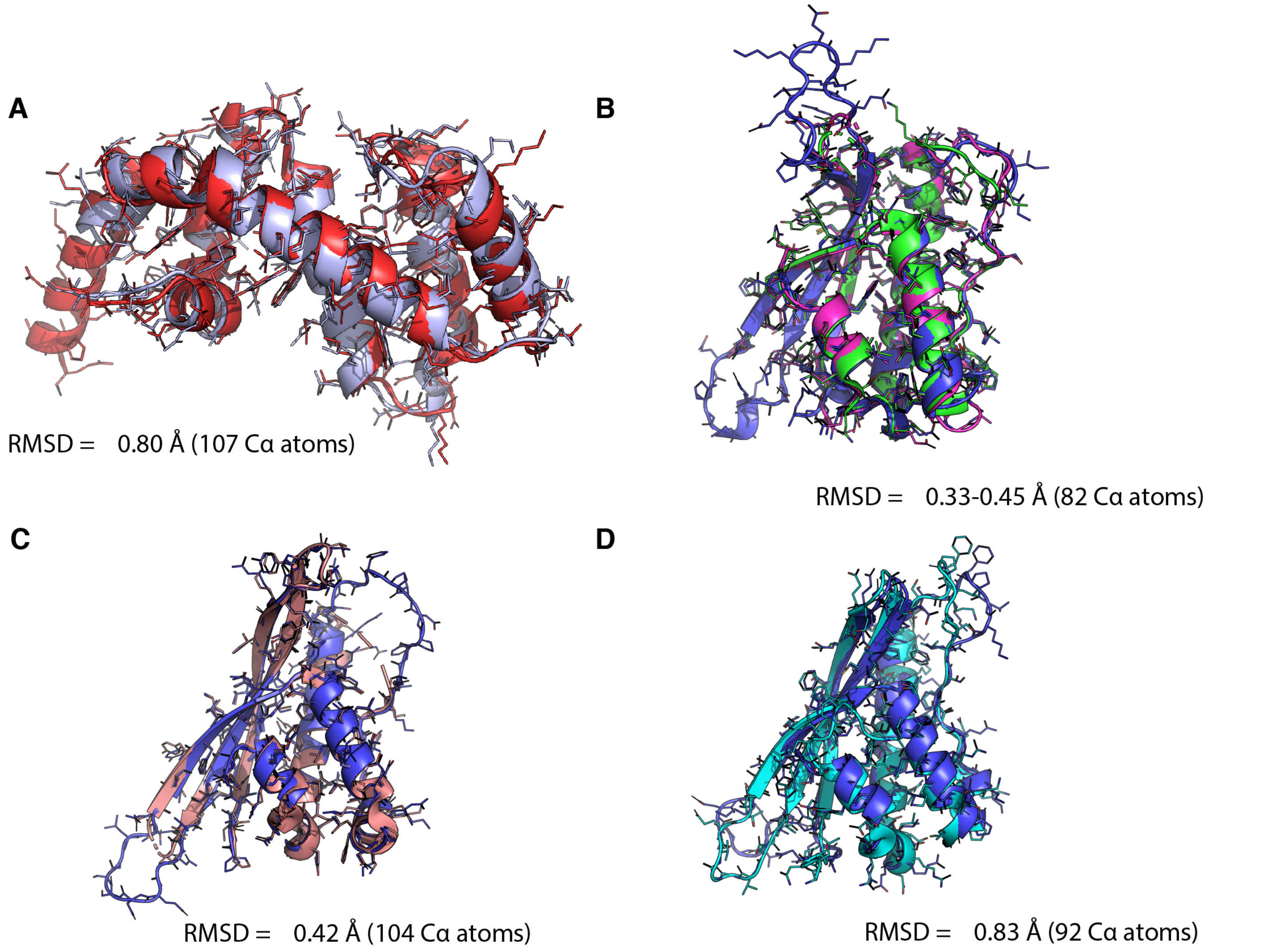

Figure S2. Comparison of experimental SNX-RGS structures with their AlphaFold2 predictions.
(A) Crystal structure of the human SNX25(C326A) RGS domain (red, this study) aligned with the AlphaFold2 prediction (light blue). (B) Crystal structures of human SNX14 PX domain (green, PDB 4PQP; magenta, 4PQO) with the AlphaFold2 prediction (blue). (C) Crystal structure of mouse SNX19 PX domain (pink, PDB 4P2I) aligned with the AlphaFold2 prediction (blue). (D) NMR structure of human SNX25 PX domain (cyan, PDB 4PQP) aligned with the AlphaFold2 prediction (blue).

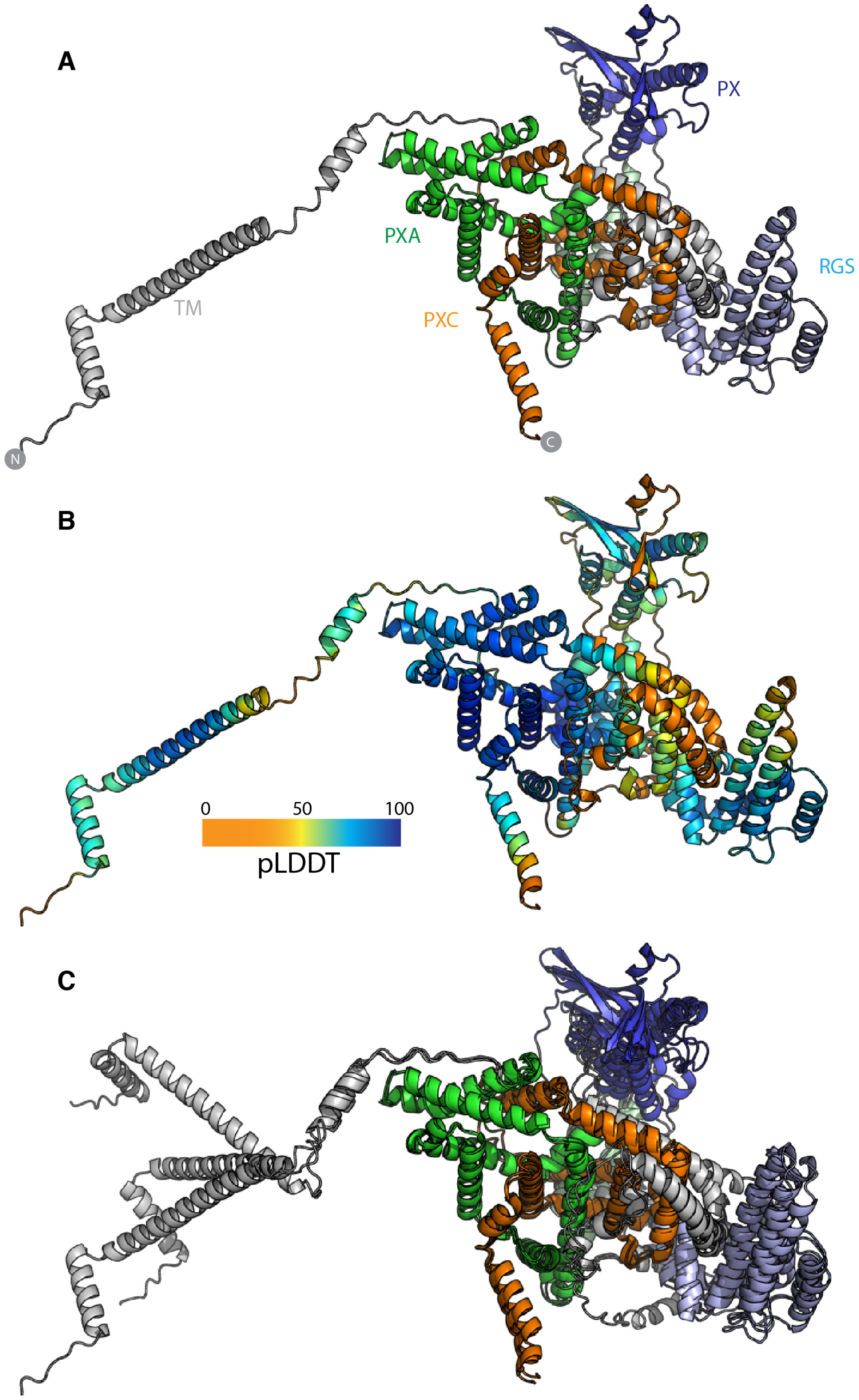

Figure S3. Additional details of the SNX13 predicted full length structure.
(A) AlphaFold2 prediction of full-length human SNX13 protein with individual domains coloured as in Fig. 2B with the TM domain and linker regions (grey), PXA domain (green), RGS domain (light blue), PX domain (blue), and PXC domain (orange). (B) AlphaFold2 prediction of full-length human SNX13 protein coloured according to the pLDDT score. (C) The AlphaFold2 predictions of human, mouse and zebrafish SNX13 proteins were aligned based on the core PXA-PXC structure.

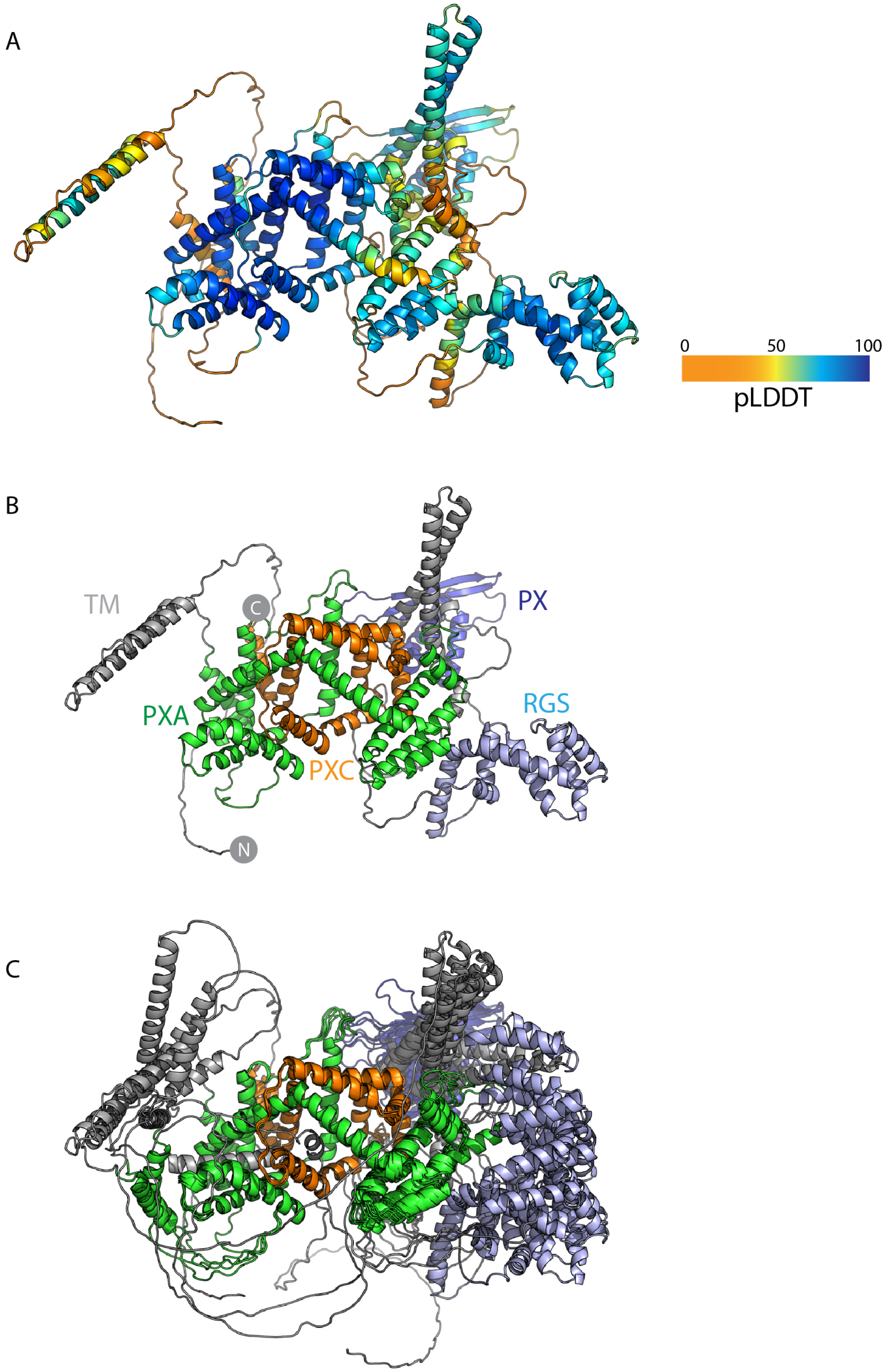

Figure S4. AlphaFold2 prediction of full-length human SNX25 protein.
(A) Top ranked structure of full length human SNX25 5 (Uniprot ID A0A494C0S0) predicted by ColabFold coloured according to pLDDT score. (B) Top ranked structure of human SNX25 predicted by ColabFold coloured according to the indicated domains. (C) Alignment based on the core PXA-PXC structure of the five structural predictions of human SNX25 from ColabFold.

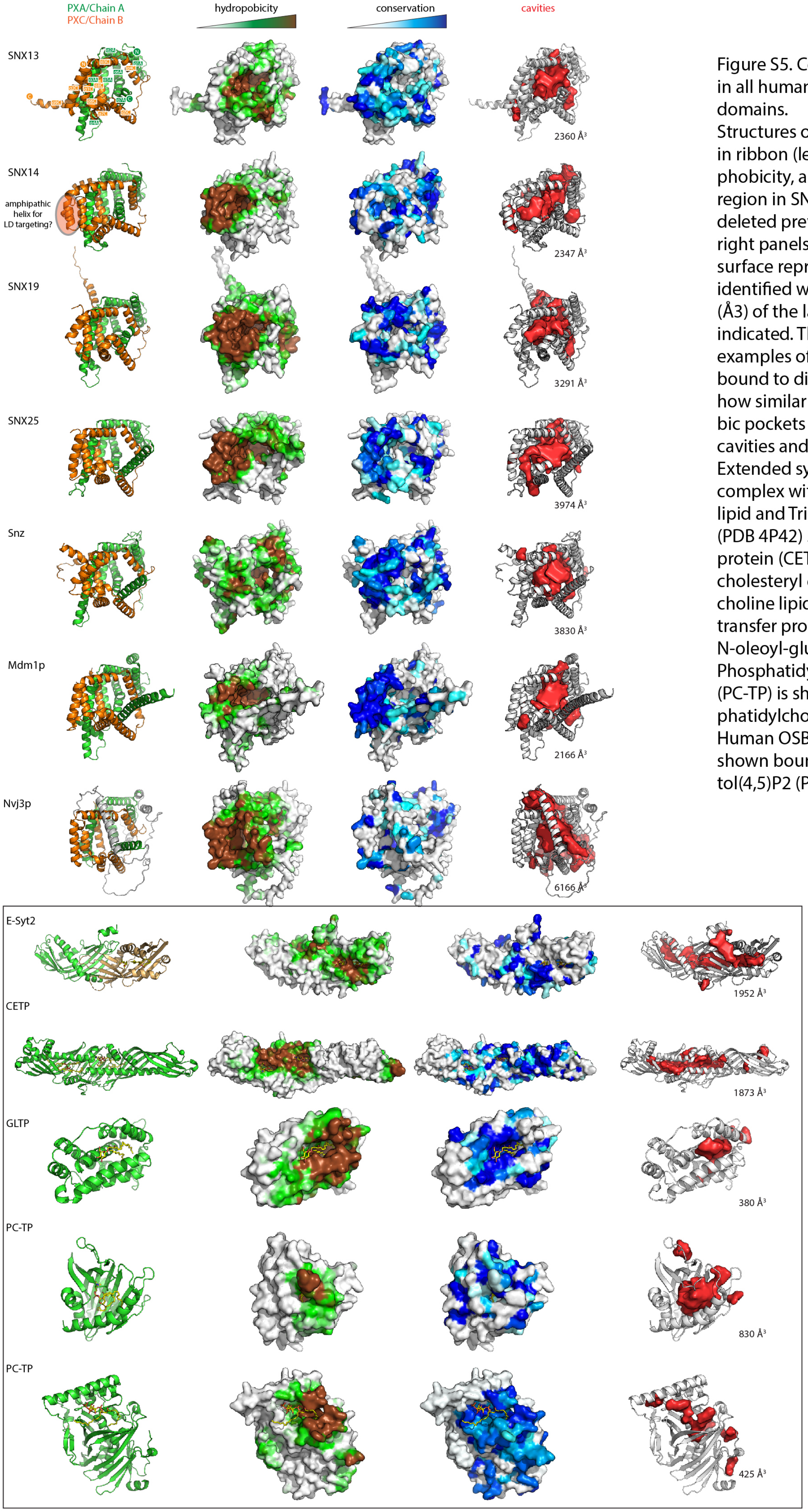

Figure S5. Conserved hydrophobic cavities in all human, yeast and fly PXA-PXC domains.
Structures of indicated PXA-PXC domains in ribbon (left) surface coloured by hydrophobicity, and sequence conservation. A region in SNX14 is highlighted that when deleted prevents LD recruitment 7. Far right panels display accessible cavities (red surface representation) in the proteins identified with POCASA56 and the volume (Å3) of the largest identified cavity is indicated. The five bottom structures are examples of other lipid transfer proteins bound to different lipids, demonstrating how similar kinds of conserved hydrophobic pockets can serve as lipid binding cavities and provided for comparison. Extended synaptotagmin 2 (E-Syt2) is in complex with a phosphatidylethanolamine lipid and Triton-X100 detergent molecule (PDB 4P42) 57. Cholesteryl ester transfer protein (CETP) is in complex with two cholesteryl esters and two phosphatidylcholine lipids (PDB 2OBP) 35. Glycolipid transfer protein (GLTP) is in complex with N-oleoyl-glucosylceramide (PDB 3S0K) 58. Phosphatidylcholine transfer protein (PC-TP) is shown in complex with a phosphatidylcholine molecule (PDB 1LN2) 59. Human OSBP-related protein 1 (ORP1) is shown bound to phosphatidylinositol(4,5)P2 (PDB 5ZM6)60

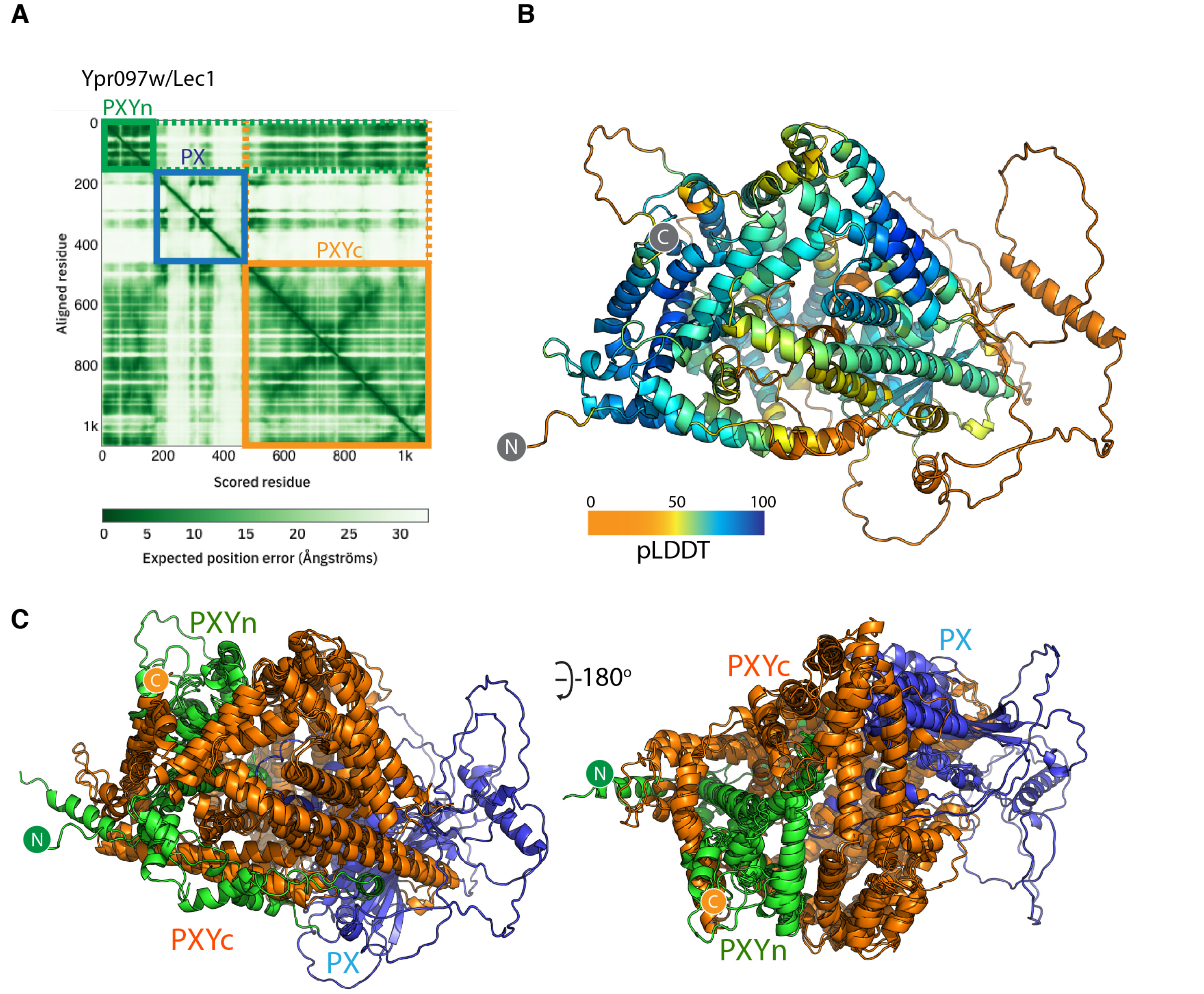

Figure S6. AlphaFold2 predicted structure of Lec1/Ypr097w.
(A) Plot of the Predicted Alignment Error (PAE) from the AlphaFold2 database. There is a strong degree of correlation between the N-terminal PXYn and C-terminal PXYc domains suggesting these two domains are physically associated. (B) The predicted structure of Lec1/Ypr097w from S. cerevisiae coloured according to the pLDDT score. (C) Overlay of the PXY proteins from S. cerevisiae, S. pombe and C. albicans.

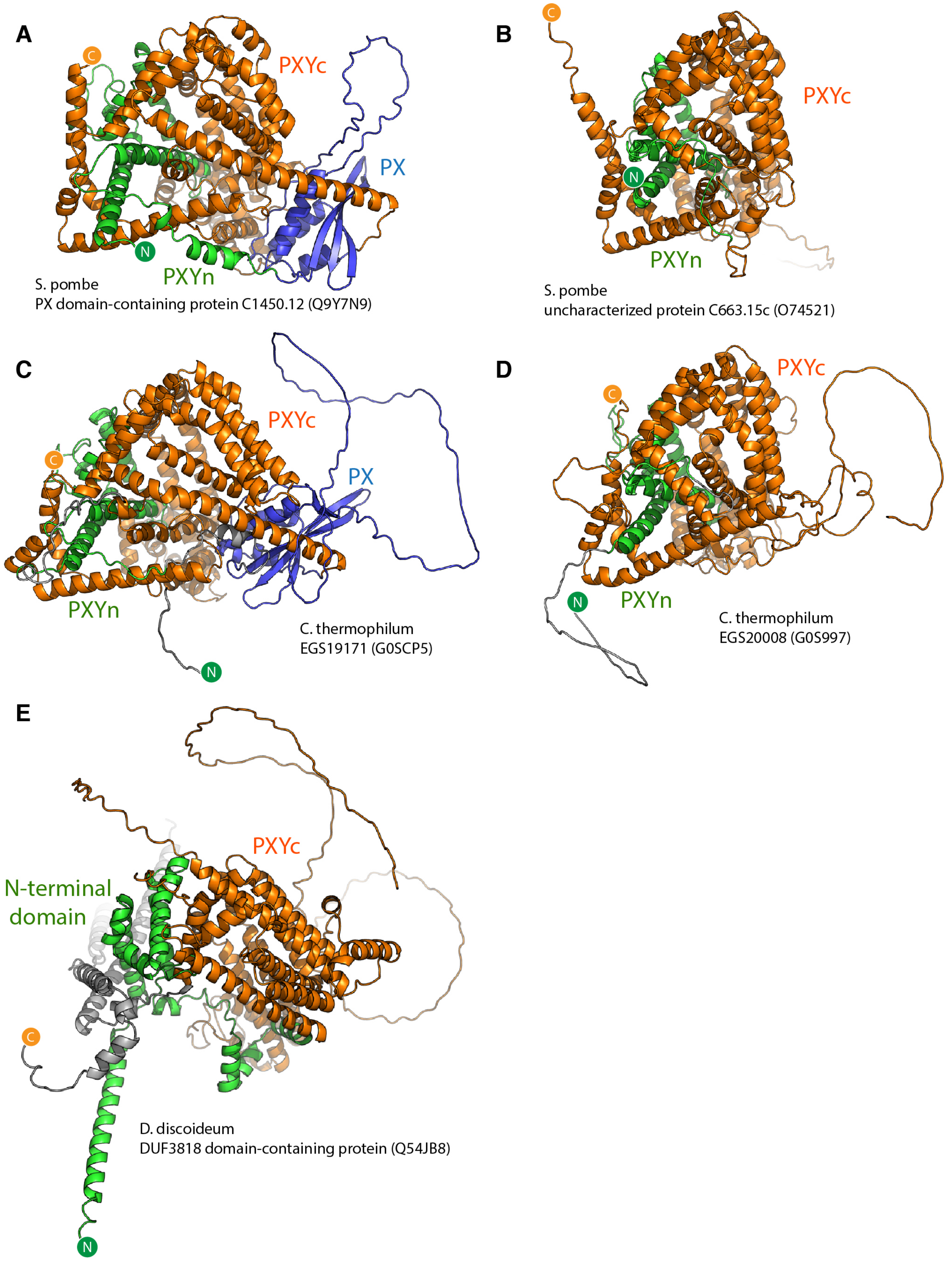

Figure S7. AlphaFold2 predicted structure of PXY domain proteins found in S. pombe, C. thermophilum and D. dictyostelium. (A) Structural prediction of S. pombe Lec1/Ypr092w orthologue C1450.12. (B) Structural prediction of S. pombe Lec1/Ypr092w homologue C663.15c. (C) Structural prediction of C. thermophilum Lec1/Ypr092w orthologue EGS19171. (D) Structural prediction of C. thermophilum Lec1/Ypr092w homologue EGS20008. (E) Structural prediction of D. discoideum DUF3818 domain-containing protein.
